# State Migration in Styx: Towards Serverless Transactional Functions

**DOI:** 10.1007/s00778-026-00971-x

**Published:** 2026-06-19

**Authors:** Kyriakos Psarakis, George Christodoulou, George Siachamis, Marios Fragkoulis, Asterios Katsifodimos

**Affiliations:** 1 Delft University of Technology, Delft, Netherlands; 2 Inria & Institut Polytechnique de Paris, Paris, France

**Keywords:** State migration, Elasticity, Serializable Transactions, FaaS

## Abstract

Developing stateful cloud applications, such as low-latency workflows and microservices with strict consistency requirements, remains arduous for programmers. The Stateful Functions-as-a-Service (SFaaS) paradigm aims to serve these use cases. However, existing approaches provide weak transactional guarantees or perform expensive external state accesses requiring inefficient transactional protocols that increase execution latency. In this paper, we present Styx, a novel dataflow-based SFaaS runtime that executes serializable transactions consisting of stateful functions that form arbitrary call-graphs with exactly-once guarantees. Styx extends a deterministic transactional protocol by contributing: i) a function acknowledgment scheme to determine transaction boundaries required in SFaaS workloads, ii) a function-execution caching mechanism, and iii) an early-commit reply mechanism that substantially reduces transaction execution latency. In addition, Styx’s elasticity supports state migration for load balancing using scale-up and scale-down operations when workloads introduce uneven overhead among workers. Experiments with the YCSB, TPC-C, and Deathstar benchmarks show that Styx outperforms state-of-the-art approaches by achieving at least one order of magnitude higher throughput while exhibiting near-linear scalability and low latency. Moreover, state migration experiments with YCSB and TPC-C show that Styx’s approach to state migration outperforms the baseline, a stop and restart migration approach tailored to Styx, by adapting swiftly to workload changes while maintaining low latency.

## Introduction

Despite the commercial availability of Functions-as-a-Service (FaaS), the model remains ill-suited for low-latency stateful applications with strict consistency requirements (e.g., payment processing and reservation systems). This limitation arises because current FaaS platforms are stateless and rely on external fault-tolerant storage systems for state management. Moreover, while workflow frameworks such as AWS Step Functions and Azure Logic Apps support function orchestration, they lack primitives for *transactional* execution. As a result, distributed applications, commonly implemented as microservices, suffer from consistency anomalies when transaction management is delegated to developers [10, 38].

Recent work argues that FaaS platforms must natively support stateful functions under the Stateful Functions-as-a-Service (SFaaS) paradigm [29, 33, 34, 57, 58, 64]. We further argue that an SFaaS runtime must (i) provide end-to-end serializable transactions across multiple functions, (ii) achieve low-latency and high-throughput execution, and (iii) expose a high-level programming model that abstracts away low-level concurrency control. To the best of our knowledge, no existing system satisfies all three requirements.

**Fig. 1:**
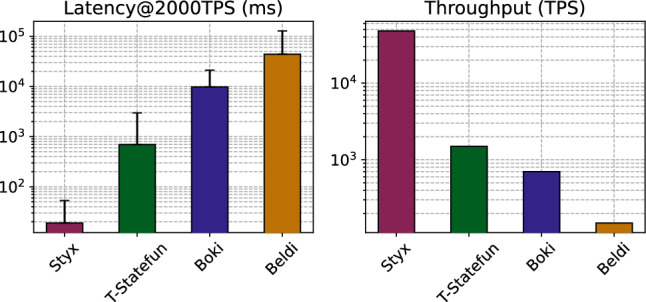
Styx outperforms state-of-the-art transactional SFaaS systems by at least one order of magnitude in transactional workloads (Section 9). Bars show median values; whiskers show 99th-percentile latency. Latency is measured at 2000 TPS; throughput corresponds to sub-second latency

State-of-the-art transactional SFaaS systems with serializable guarantees, including Boki [33], Beldi [64], and T-Statefun [29], support transactional workflows but incur high commit latency and low throughput. Their inefficiency stems from separating state storage from function execution and relying on locking and Two-Phase Commit (2PC) to coordinate cross-function transactions.

This paper proposes Styx, a dataflow-based runtime for SFaaS that provides exactly-once processing and end-to-end serializable transactions across arbitrary function orchestrations, even in the presence of failures and retries. Styx leverages deterministic transaction execution to avoid 2PC, enabling early commit replies before transaction snapshots are durably persisted.

Our design is motivated by two observations. First, modern streaming dataflow systems such as Apache Flink [7, 8, 55] provide exactly-once guarantees through transparent failure handling, but cannot execute general cloud applications or transactional SFaaS workflows. Second, deterministic database protocols [44, 61] avoid costly coordination but do not support complex function orchestrations or arbitrary call graphs. Styx bridges this gap by integrating deterministic transactions into a dataflow execution model.

Styx relates to recent proposals such as DBOS [56], Hydro [11], and serverless storage-management systems (SSMSs) [43]. In contrast, Styx adopts a streaming dataflow execution model and guarantees serializability *across* functions. As shown in Figure 1, Styx achieves one order of magnitude lower median latency, up to two orders of magnitude lower tail latency, and one order of magnitude higher throughput than existing serializable SFaaS systems.

Styx was introduced and evaluated in the original version of this paper [50]. While its internals are already transparent to developers—requiring no transactional or fault-tolerance code—operational concerns such as resource management remain visible. This work addresses that gap by adding elastic state migration, a prerequisite for a fully serverless SFaaS runtime. Styx migrates fine-grained state at key-set granularity, either on demand for active transactions or asynchronously for inactive keys.Unlike prior VM-based migration techniques [12, 30], our approach preserves transactional guarantees while tightly coupling state movement with function execution.

**Contributions:**

- We design Styx, a dataflow-based SFaaS runtime that integrates deterministic transactions with streaming execution (Section 2).
- We present a high-level SFaaS programming model that abstracts away transaction management and failure handling while guaranteeing exactly-once processing and serializability across arbitrary function calls (Sections 3–6).
- We extend deterministic database techniques to support complex function orchestrations, contributing a novel acknowledgment scheme and execution-caching mechanism (Sections 5.3, 6.3).
- We enable early commit replies through deterministic execution, allowing transactions to complete before durable snapshot persistence (Section 6.4).
- We show that Styx outperforms state-of-the-art transactional SFaaS systems by at least one order of magnitude in throughput while achieving lower latency and near-linear scalability (Section 9).
- We introduce an elastic state-migration mechanism that enables near-zero-downtime scaling while preserving transactional guarantees (Sections 8, 9.5).

Styx is available at: https://github.com/delftdata/styx

## Motivation

In this section, we analyze the specifics of streaming dataflow system design and argue that they can be extended to encapsulate the primitives required to consistently and efficiently execute workflows of stateful functions. Our work is based on a key observation: the architecture of high-performance cloud services closely resembles a parallel dataflow graph, in which state is partitioned and co-located with the application logic [49]. As argued in [49], the evolution from monolithic applications to microservices and Function-as-a-Service (FaaS) architectures has shifted responsibility for transaction management, fault tolerance, and state consistency from the database to the application layer. In monolithic systems, developers focused primarily on business logic, while a transactional database provided atomicity, isolation, and recovery. In contrast, modern cloud applications decompose functionality into loosely coupled services that communicate via asynchronous messaging and maintain partitioned state. Although this architectural style improves modularity and scalability, it requires developers to manually address idempotency, message retries, workflow orchestration, and cross-service consistency, often through ad hoc mechanisms such as Sagas or two-phase commit, which either weaken guarantees or degrade performance.

The key insight of [49] is that these distributed service architectures already implicitly implement a streaming dataflow graph: services correspond to operators, communication channels correspond to streams, and state is naturally partitioned and co-located with compute. Modern stream processing systems provide exactly-once processing, consistent snapshot-based fault tolerance, data-parallel scale-out, and high-throughput execution. However, they are traditionally designed to support analytics workloads and lack transactional semantics across arbitrary workflows of stateful functions. By combining the execution model of stateful streaming dataflows with deterministic transactional protocols, it becomes possible to execute complex, nested workflows with serializable guarantees, without relying on blocking coordination or compensating actions. Determinism aligns naturally with dataflow replay and checkpointing, enabling failure recovery without rollbacks and allowing low-latency commit decisions.

Therefore, streaming dataflow systems provide a principled systems substrate for transactional cloud applications: they unify messaging, state management, orchestration, and fault tolerance within a single runtime, restoring database-like guarantees while preserving the scalability and modularity of cloud-native architectures.

Additionally, as we detail in Section 2.2, there is a synergy between deterministic transactions and dataflow systems. Such a combination can provide state consistency and ease of programming, as monolithic solutions did in the past, while improving scalability and reducing developer involvement. Finally, we show how deterministic transactions can be extended for SFaaS, where transaction boundaries are unknown, unlike online transaction processing (OLTP).

### Dataflows for Stateful Functions

Stateful dataflows are the execution model implemented by virtually all modern stream processors [8, 46, 48]. In addition to being a strong fit for parallel, data-intensive computations, stateful dataflows are the primary abstraction supporting workflow managers such as Apache Airflow [28], AWS Step Functions [63], and Azure’s Durable Functions [6]. In the following, we present the primary motivation behind using stateful dataflows to build a suitable runtime for orchestrating general-purpose cloud applications.

**Exactly-once Processing** Message-delivery guarantees are fundamentally hard to deal with in the general case, with the root of the problem being the well-known Byzantine Generals problem [39]. However, in the closed world of dataflow systems, exactly-once processing is possible [7, 8, 55]. In fact, the APIs of popular streaming dataflow systems, such as Apache Flink, require no error-management code (e.g., message retries or duplicate elimination using idempotency IDs).

**Co-Location of State and Function** The primary reason streaming dataflow systems can sustain millions of events per second [8, 25] is that their state is partitioned across operators that operate on local state. While the structure of current Cloud offerings favors the disaggregation of storage and computation, we argue that co-locating state and computation is the primary means of achieving high performance and can also be adopted by modern SFaaS runtimes, rather than relying on external databases for state storage.

**Coarse-Grained Fault Tolerance** To ensure atomicity at the level of workflow execution, existing SFaaS systems perform fine-grained fault tolerance [33, 64]; each function execution is logged and persisted in a shared log before the next function is called. This requires a round-trip to the logging mechanism for each function call, which adds significant latency to function execution. Instead of logging each function execution, streaming dataflow systems [7, 9, 54] employ a coarse-grained fault tolerance mechanism based on asynchronous snapshots, thereby reducing this overhead.

### Determinism & Transactions

Given a set of database partitions and a set of transactions, a deterministic database [2, 60] will end up in the same final state despite node failures and possible concurrency issues. Traditional database systems offer *serializable* guarantees, allowing multiple transactions to execute concurrently, ensuring that the database state will be equivalent to the state of one serial transaction execution. Deterministic databases guarantee not only serializability but also that a given set of transactions will have exactly the same effect on the database state, even after re-execution. This guarantee has important implications [2] that have not yet been leveraged by SFaaS systems.

**Deterministic Transactions on Dataflows** Unlike 2PC, which requires rollbacks in case of failures, deterministic database protocols [44, 61] are "forward-only": once the locking order [61] or read/write set [44] of a batch of transactions has been determined, the transactions are going to be executed and reflected on the database state, without the need to rollback changes. This notion is in line with how dataflow systems operate: events flow through the dataflow graph, from sources to sinks, without stalls for coordination. This match between deterministic databases and the dataflow execution model is the primary motivation behind Styx’s design choice to implement a deterministic transaction protocol on top of a dataflow system.

### Challenges

Despite their success and widespread applicability, dataflow systems require multiple modifications before they can be used for transactional, stateful functions. In the following, we list challenges and open problems tackled in this work.

**Programming Models** Dataflow systems at the moment are only programmable through functional programming-style dataflow APIs: a given cloud application has to be rewritten by programmers to match the event-driven dataflow paradigm. Although it is possible to rewrite many applications in this paradigm, it takes a considerable amount of programmer training and effort. We argue that dataflow systems would benefit from object-oriented or actor-like programming abstractions in order to be adopted for general cloud applications, such as microservices.

**Support for Transactions** In the context of streaming dataflow systems, transactions typically refer to processing a set of input elements and their state updates with ACID guarantees [65]. Despite progress, critical challenges remain, such as the performance overhead of multi-partition transactions and the need to block data flows for locking and message reordering. In this work, we argue that, when implementing transactions in a streaming dataflow system, we need to "keep the data moving" [59] by avoiding disruptions to the natural flow of data while tightly integrating transaction processing into the system’s state management and fault tolerance protocols.

**Deterministic OLTP and SFaaS** OLTP databases that use deterministic protocols like Calvin [44, 61] either require each transaction’s read/write set a priori or are extended to discover the read-write sets of a transaction by first executing it. Additionally, in both scenarios, deterministic protocols assume that a transaction is executed as a single-threaded function that can perform remote reads and writes from other partitions. In the case of SFaaS, arbitrary function calls enable programmers to leverage both the separation-of-concerns principle, widely applied in microservice architectures [38], and code modularity. Although deterministic database systems have been proven to perform exceptionally well [2], designing and implementing a deterministic transactional protocol for arbitrary workflows of stateful functions is non-trivial. Specifically, arbitrary function calls create complex call-graphs that need to be tracked in order to establish a transaction’s boundaries before committing.

**Dataflows for Arbitrary-Workflow Execution** The prime use case for dataflow systems nowadays is streaming analytics. However, general-purpose cloud applications have different workload requirements. Functions calling other functions and receiving responses introduce cycles in the dataflow graph. Such cycles can cause deadlocks and must be handled [40].

In this work, we address these challenges and propose a dataflow system tailored to stateful functions, with built-in support for deterministic transactions and a high-level programming model.

## Programming Model

**Fig. 2:**
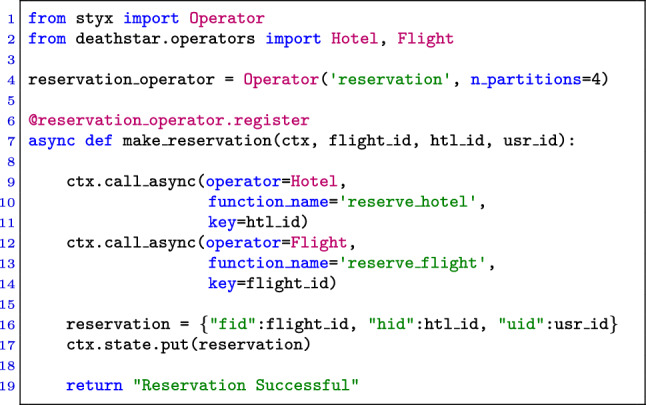
Deathstar’s[24] Hotel/Flight reservation in Styx. From lines 9-14, the $reserve\_hotel$ and $reserve\_flight$ functions are invoked asynchronously. Finally, in lines 16-17, the reservation information is stored. In Styx, the transactional and fault tolerance logic are handled internally

The programming model of Styx is based on Python and comprises operators that encapsulate partitioned mutable state and functions that operate on that. An example of the programming model of Styx is depicted in Figure 2.

### Programming Model Notions

**Stateful Entities** Similar to objects in object-oriented programming, entities in Styx are responsible for maintaining and mutating their own state. Moreover, when a given entity needs to update another entity’s state, it can do so via a function call. Each entity bears a unique, immutable key that is application-dependent and contains no information about its physical location. The dataflow runtime engine (Section 4) uses that key to route function calls to the right operator that accommodates that specific entity.

**Functions** functions can mutate the state of an entity. By convention, the context is the first parameter of each function call. Functions are allowed to call other functions directly, and Styx supports both synchronous and asynchronous function calls. For instance, in lines 9-11 of Figure 2, the instantiated reservation entity will call asynchronously the function ’reserve_hotel’ of an entity with key ’hotel_id’ attached to the Hotel operator.

**Fig. 3:**
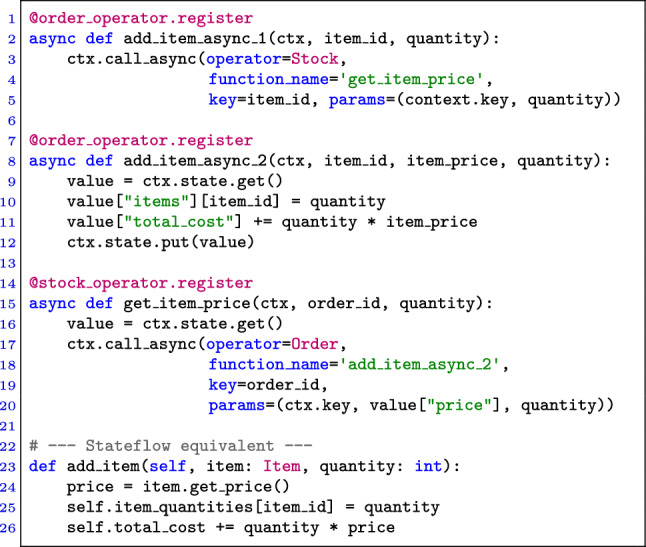
Shopping cart example in Styx with async continuations that can be abstracted with Stateflow [51]

Similarly, one could issue a synchronous call that blocks until the result becomes available. However, such blocking behavior would be detrimental to performance in a distributed, event-driven setting. Instead, using the Styx API, developers can explicitly structure an asynchronous workflow using custom continuations. Figure 3 illustrates this pattern for the task of adding an item to an order. The entry-point function, add_item_async_1, asynchronously invokes get_item_price on the Stock operator to retrieve the item’s price. Rather than returning a value directly, this call supplies the order_id as a callback key. Once the price is retrieved, get_item_price issues a second asynchronous call that invokes add_item_async_2 on the Order operator using the callback key. This second function resumes the logical continuation of the original operation by updating the order state with the retrieved price and quantity. Together, these two functions manually encode a split-phase asynchronous workflow that emulates a synchronous function call. While this approach is fully asynchronous and avoids blocking, it requires developers to manage control flow, callbacks, and intermediate state explicitly, which can be challenging to write and reason about. This pattern is used in our implementation of the TPC-C experiment (Section 9), but it highlights the usability challenges of low-level asynchronous programming.

This motivates the second option: expressing the same logic using the higher-level Stateflow [51] API, which is supported by Styx. Stateflow allows developers to write code that appears synchronous, as shown in lines 23–26 of Figure 3, while the system transparently compiles it into an equivalent asynchronous execution. In that way, Stateflow eliminates the need for manually defined continuations without sacrificing performance or scalability.

**Operators** Each entity directly maps to a dataflow operator (also called a vertex) in the dataflow graph. When an *event* enters the dataflow graph, it reaches the operator that holds the *function code* of the given entity and its *state*. In short, a dataflow operator can execute all functions of a given entity and store its state. Since operators can be partitioned across multiple cluster nodes, each partition stores a set of stateful entities indexed by their unique key. When an entity’s function is invoked (via an incoming event), the entity’s state is retrieved from the local operator state. Then, the function is executed with the arguments from the incoming event that triggered the call.

**State & Namespacing** As mentioned before, each entity has access only to its own state. In Styx, the state is *namespaced* with respect to the entity it belongs to. For instance, a given key "hotel53" within the operator Hotel is represented as: entities://Hotel/hotel53. This way, a reference to a given key of a state object is unique and can be determined at runtime when operators are partitioned across workers. Programmers can store or retrieve state through the context object by invoking context.put() or get() (e.g., in Line 17 of Figure 2). Styx’s context is similar to the context object used in other systems such as Flink Statefun, AWS Lambda, and Azure Durable Functions.

**Transactions** A transaction in Styx corresponds to the execution of an entire stateful function call-graph, initiated by a client request and executed as a single atomic unit with serializable isolation and exactly-once semantics. From the programmer’s perspective, the transaction boundary is defined by the function call that specifies the transactional workflow. Internally, however, Styx performs concurrency control and conflict detection at a finer granularity: individual state read and write operations on keys. To this end, Styx tracks all state accesses performed during the execution of a function call-graph and constructs a Read/Write (RW) set for each transaction. Conflicts are defined at the key level: two concurrent transactions conflict if one transaction reads or writes a key that the other transaction has written. Each function call-graph is assigned a unique transaction identifier (TID), and conflicting transactions are serialized according to their assigned order. Moreover, Styx’s programming model allows transaction aborts by raising an uncaught exception. In the example of Figure 2, if a hotel entity does not have enough availability when calling the *’reserve_hotel’* function, the *’make_reservation’* transaction should be aborted, alongside potential state mutations that the *’reserve_flight’* has made to a flight entity. In that case, the programmer has to raise an exception as follows:
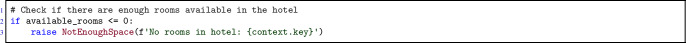


The exception is caught by Styx, which automatically triggers the transaction’s abort/rollback sequence and sends the user-defined exception message as a reply.

**Exactly-once Function Calling** Styx offers *exactly-once processing* guarantees: it reflects the state changes of a function call execution exactly-once. Thus, programmers need not “pollute” their application logic with consistency checks, state rollbacks, timeouts, retries, and idempotency [36, 38]. We detail this capability in Section 7.

## Styx’s Architecture

**Fig. 4:**
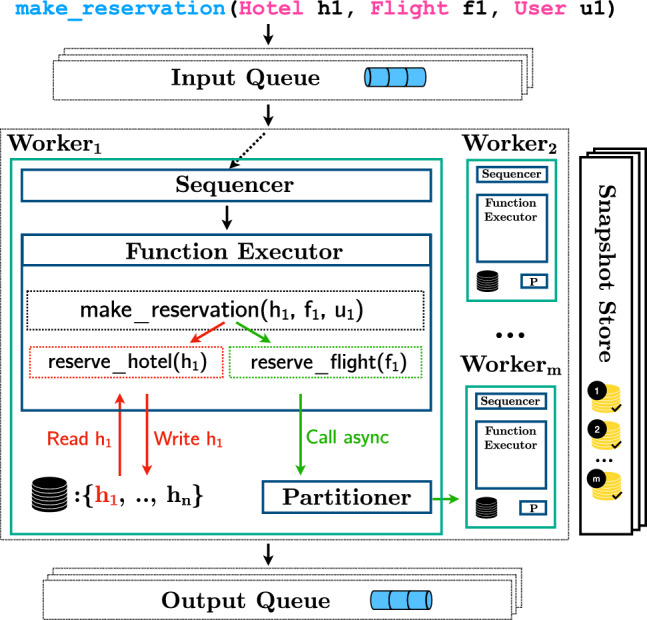
Stateful-function execution in Styx. Each worker uses one coroutine to sequence incoming transactions and another to execute them. In this example, the make_reservation transaction invokes reserve_hotel (local state access) and reserve_flight (a remote call to another partition, located via the partitioner)

In this section, we describe the components (Figure 4) and the main design decisions of Styx.

### Components

**Coordinator** The coordinator manages and monitors Styx’s workers and the cluster runtime state (transactional metadata, dataflow state, partition locations, etc.). It also performs scheduling and health monitoring. Styx monitors the cluster’s health using a heartbeat mechanism and initiates the fault-tolerance mechanism (Section 7) once a worker fails.

**Worker** As depicted in Figure 4, the worker is the primary component of Styx, processing transactions, receiving or sending remote function calls, and managing state.

The worker consists of two primary coroutines. The first coroutine ingests messages for its assigned partitions from a durable queue and sequences them. The second coroutine receives a sequence of transactions and initiates transaction processing. By using the coroutine execution model, Styx improves efficiency, since the primary source of latency is waiting for network or state-access calls. Coroutines enable single-threaded concurrent execution, switching between coroutines when one is suspended during a network call, allowing others to make progress. Once the network call is completed, the suspended coroutine resumes processing.

**Fig. 5:**
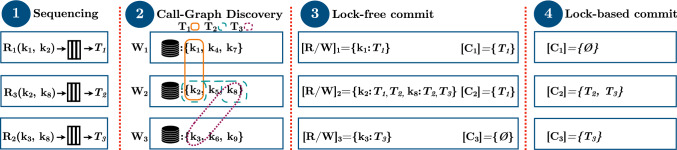
The transaction execution pipeline in Styx is divided into 4 parts. First, each external request ($R_i$) is sequenced as a transaction and is assigned a unique id. Afterward, the transactions execute their application logic, accessing local keys and performing remote function calls. While a transaction executes, Styx tracks its accessed keys ($[R/W]_i$) and incrementally constructs its call-graph. Subsequently, Styx commits the transactions that do not participate in unresolved conflicts without having to perform locking. For example, we observe that workers $W_{1}$ and $W_{2}$ are capable to commit $C_1 = C_2 = \bigl \{T_1\bigr \}$ while $T_{1}$ interacts with the same keys as $T_{2}$; although it has the lowest id. In the final part, we commit all the transactions by resolving the conflicts with a lock-based mechanism ($C_2 = \bigl \{T_2, T_3\bigr \}$), $C_3 = \bigl \{T_3\bigr \}$

**Partitioning Stateful Entities Across Workers** Styx makes use of the entities’ key to distribute those entities and their state across several workers. By default, each worker is assigned a set of keys using hash partitioning.

**Input/Output Queue** For fault tolerance, Styx assumes a persistent input queue from which it receives requests from external systems (e.g., from a REST gateway API). Styx requires that the input queue be able to deterministically replay messages at a specified offset upon failure. As we detail in Section 7, the replayable input queue is necessary for Styx to produce the same sequence of transactions after the recovery is complete and to enable early commit-replies (6.4). In the same way, Styx sends the result of a given transaction to an output queue from which an external system (e.g., the same REST gateway API) can receive it. Currently, Styx leverages Apache Kafka [37].

**Durable Snapshot Store** Alongside the replayable queue, durable storage is necessary for storing the workers’ snapshots. Currently, Styx uses Minio, an open-source S3-compatible service, to store the incremental snapshots as binary files.

### Transaction Execution Pipeline

Styx employs an epoch-based transactional protocol that concurrently executes a batch of transactions in each epoch. A transaction may include multiple functions that, during runtime, form a call-graph of function invocations. Each function may mutate its entity’s state, and the effects of function invocations are committed to the system state in a transactional manner. In Figure 4, once make_reservation enters the system, it is persisted and replicated by the input queue. Then, a worker ingests the call into its local sequencer, which assigns a Transaction ID (TID) and processes all encapsulated function calls as a single transaction. In the make_reservation case, the transaction consists of two functions: reserve_hotel and reserve_flight. For this example, let us assume that reserve_hotel is a local function call and reserve_flight runs on a remote worker. reserve_hotel will execute locally asynchronously using coroutines and apply state changes. In contrast, reserve_flight will execute asynchronously on a remote worker, applying changes to the remote state.

**Fig. 6:**
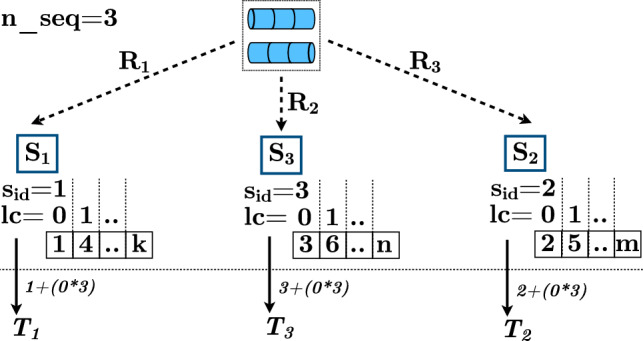
Example of TID assignment in Styx with three sequencers. Their identifiers $\{1, 2, 3\}$ lead to the following sequences: $S_1 = \{1, 4,..., k\}$, $S_2 = \{2, 5,..., m\}$, $S_3 = \{3, 6,..., n\}$ following the formula expressed in Equation 1

## Sequencing & Function Execution

The deterministic execution of functions with serializable guarantees requires a sequencing step that assigns a transaction ID (TID), which, in combination with the read/write (RW) sets, can be used for conflict resolution (Section 6). The challenge we tackle in this section is determining the boundaries of transactions (i.e., when a transaction’s execution begins and ends), which arises from the execution of arbitrary function call-graphs (Section 5.3).

### Transaction Sequencing

In this section, we discuss the sequencing mechanism () of Styx. Deterministic databases ensure the serializable execution of transactions by forming a global sequence. In Calvin [61], the authors propose a partitioned sequencer that retrieves the global sequence by communicating across all partitions, performing a deterministic round-robin.

**Eliminating Sequencer Synchronization** Instead of the original sequencer of Calvin that sends $\mathcal {O}(n^2)$ messages for the deterministic round-robin, Styx adopts a method similar to the one followed by Mencius [45], allowing Styx to acquire a global sequence without any communication between the sequencers ($\mathcal {O}(1)$). This is achieved by having each sequencer assign unique transaction identifiers (TIDs) as follows:

1 $$\begin{aligned} TID_{sid, lc} = sid + (lc * n\_seq) \end{aligned}$$

where $sid \in \mathbb {N}_1$ is the sequencer id assigned by the Styx coordinator in the registration phase, $lc \in \mathbb {N}_0$ is a local counter of each sequencer specifying how many TIDs it has assigned thus far, and $n\_seq \in \mathbb {N}_1$ is the total number of sequencers in the Styx cluster. In the example of Figure 5, the sequencers of the three workers will sequence $R_1$, $R_2$ and $R_3$ to $T_1$, $T_3$ and $T_2$ respectively. Figure 6 illustrates how those TIDs are generated in parallel. Note that, conceptually, Styx implements a partitioned sequencer where the global sequence $S = \{S_1 \cup S_2 \cup \dots \cup S_n\}$ is the union of all partitioned sequences.

**Mitigating Sequence Imbalance** In case a single sequencer $S_1$ receives more traffic than the other sequencers, its local counter ($lc_1$) will increase more than the local counter of the rest of the sequencers. As a result, in the next epoch, sequencer $S_1$ would produce larger TIDs than the rest of the sequencers. This means that new transactions arriving at a less busy sequencer will receive higher execution priority: transactions with higher TID receive lower priority in our transactional protocol. In the event of high contention in the workload, this would increase latency for the busy ($S_1$) worker node. To avoid this, at the end of each epoch, the coordinator computes the maximum *lc* ($max(lc_1, lc_2, \ldots , lc_n)$) and communicates it to all workers so that they can adjust their local counters and rebalance sequences in each epoch. Balancing workers’ transaction priorities reduces 99th-percentile latency.

**Replication and Logging** There is no need to replicate and log the sequence within Styx since the input is logged and replicated within the replayable queue. In case of failure, after transaction replay, the sequencers will produce the exact same sequence (Section 7.2).

### Call-Graph Discovery

After sequencing, Styx needs to execute the sequenced transactions and determine their call-graphs and RW sets (). To this end, the function execution runtime ingests a given sequence of transactions to process in a given epoch. The number of transactions per epoch is either specified by a time interval (by default, 1 millisecond) or by a configurable maximum number of transactions (by default, 1000). We have chosen an epoch-based approach since processing the incoming transactions in batches increases throughput.

Styx’s runtime executes all the sequenced transactions on a snapshot of the data to discover the read/write sets. Transactions that span multiple workers will implicitly change the read/write sets of the remote workers via function calls. There is an additional issue related to discovering the RW set of a transaction: before the functions execute, the call-graph of the transaction is unknown. This is problematic because the protocol requires that all transactions be completed before proceeding to the next phase. To tackle this problem, Styx proposes a function acknowledgment scheme explained in more detail in Section 5.3.

After this phase, all stateful functions comprising transactions will have completed execution, and the RW sets will be known. In Figure 5, transactions $T_1$, $T_2$, and $T_3$ will execute and create the following RW sets: $Worker_1 \rightarrow \{k_1: T_1\}$, $Worker_2 \rightarrow \{k_2: T_1,T_2$ and $k_8: T_2, T_3\}$ and $Worker_3 \rightarrow \{k_3: T_3\}$.

**Fig. 7:**
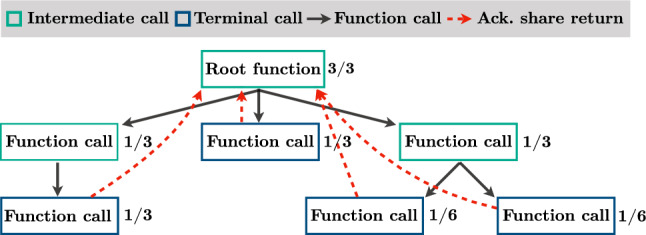
Asynchronous function call chains. A root function may invoke other functions during execution. Its acknowledgment (3/3) is progressively split into ack-shares, with each callee receiving a portion. In this example, the root function calls three functions and divides its ack-share equally among them; subsequent calls split their shares similarly. The ack-shares of all terminal (blue) calls sum to exactly 3/3, allowing the root function to detect completion

### Function Execution Acknowledgment

In the SFaaS paradigm, the call-graph formed by a transaction is unknown; functions could be coded by different developer teams and can form complex call-graphs. This uncertainty complicates determining when a transaction has completed processing, which is essential because phase  can only start after all transactions have finished processing. To that end, each asynchronous function call of a given transaction is assigned an ack_share. A given function knows how many shares to create by counting the number of asynchronous function calls during its runtime. The caller function then sends the respective acknowledgment shares to the downstream functions. For instance, in Figure 7, the transaction entry-point (root of the tree) calls three remote functions, splitting the ack_share into three parts (3 x $1/3$). The left-most function invokes only one other function and passes to it its complete ack_share ($1/3$). The middle function does not call any functions, so it returns the share to the root function when it completes execution, and the right-most function calls two other functions, splitting its share ($1/3$) to 2 x $1/6$. After all the function calls are complete, the root function should have collected all the shares. When the sum of the received shares adds to 1, the root/entry-point function can safely deduce that the execution of the complete transaction is complete. This design is devised for two reasons: i) if every participating function just sent an ack upon completion, the root would unable to calculate the expected acks ensuring that the entire execution has finished, and ii) with fractions we avoid the well-known challenge related to adding floating point numbers.

The ack_shares are agnostic to the application code, even in the case of branching. For example:
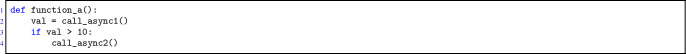


In this example, the amount of ack_shares is determined by the val. However, the number of shares is computed at runtime once function_a completes; whether the condition is met or not, it does not affect the process. The more call_async calls happen, the more slices of the share function_a will create, and the outcome will remain the same. The root function still waits until all slices of terminal calls sum to 1.

A solution close to the ack_share is one of the distributed futures [62]. However, it would not work in the SFaaS context, as it either requires information about the entire call-graph to operate asynchronously or requires creating a chain of futures, thereby making it synchronous. Hence, it would introduce high latency for our use case.

## Committing Transactions

After completing an epoch’s call-graph discovery, Styx needs to determine which transactions will commit and which will abort based on the transactions’ Read/Write (RW) sets and TIDs. To this end, this section presents two different commit phases: *i*) an optimistic lock-free phase that commits only the non-conflicting transactions, and *ii*) a lock-based phase that only commits the transactions that were not able to commit in the first phase. The lock-based commit phase commits all conflicting transactions by acquiring locks in a TID-ordered sequence. To accelerate the second phase, we have devised a caching scheme that reuses the previously discovered call-graph to avoid re-executing long function chains whenever possible (Section 6.3).

### Lock-free Commit Phase

In case of conflict (i.e., a transaction *t* writes a key that another transaction $t'$ also reads or writes on), similarly to [44], only the transaction with the lowest transaction ID will succeed to commit (). Uncommitted transactions are placed in a queue for execution in the next phase  (while retaining their previously assigned IDs).

In addition, workers (*W*) send their local conflicts to every other worker via the coordinator ($2*|W|$ messages). This ensures that every worker maintains a global view of all aborted/rescheduled transactions and can, locally, decide which transactions can be committed. Finally, note that transactions can also abort not because of conflicts but because of application logic (e.g., by throwing an exception). In that case, Styx removes the corresponding entries from the read/write sets to reduce potential conflicts further.

In this phase, all transactions that are not part of a conflict apply their writes to the state, commit, and respond to clients. In the example shown in Figure 5, only $T_1$ can commit in $W_1$ and $W_2$ due to conflicts in the RW sets of $W_2$ regarding $T_2$ and $T_3$; more specifically, at keys $k_2$ and $k_8$.

### Lock-based Commit Phase

In the previous phase, , only transactions without conflicts can be committed. We now explain how Styx deals with transactions that have not been committed in a given epoch due to conflicts (). First, Styx acquires locks in a given sequence ordered by transaction ID. Then it reruns all transactions concurrently, since all read/write sets are known, and commits them. However, if a transaction’s read/write set changes in this phase, Styx aborts the transaction and recomputes its read/write set in the next epoch. Now, in Figure 5, $W_2$ can sequentially acquire locks for $T_2$ and $T_3$, leading to their commits in $W_2$ and $W_3$.

### Call-Graph Caching

As depicted in Figure 5, the lock-based commit phase  is used to execute any transactions that did not commit during the lock-free commit phase . By the time the lock-based commit phase begins, the database state may have changed since the lock-free commit. As a result, function invocations must be re-executed to reflect the data updates.

**Fig. 8:**
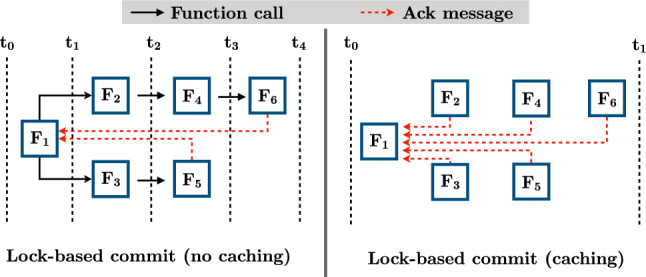
If no function caching is performed (left), the transaction execution will execute a deep call-graph; the messages will be sent sequentially and be equal to the number of function calls (5) in addition to the acks (2). Styx’s function caching optimization (right) will lead to a concurrent function execution in the lock-based commit phase, between $t_0$ and $t_1$, and send only five acks asynchronously

On the left part of Figure 8, we depict such a function invocation. At time $t_0$, F$_1$ is invoked, which in turn invokes two function chains: $F_1 \rightarrow F_2 \rightarrow F_4 \rightarrow F_6$ and $F_1 \rightarrow F_3 \rightarrow F_5$. Once the two function chains finish their execution (on time $t_4$ and $t_3$ respectively), they can acknowledge their termination to the root call $F_1$.

**Potential for Caching** During our early experiments, we noticed cases where $F_1$ is invoked and the parameters with which it calls $F_2$ (and in turn the invocations across the $F_1 \rightarrow ... \rightarrow F_6$ call chain) do not change. The same applied to the RW sets of those function invocations; they remained unchanged. Since Styx tracks those call parameters as well as the functions’ RW sets, it can cache input parameters during the lock-free commit phase and reuse them during the lock-based commit, avoiding long sequential re-executions along the call chains. This case is depicted on the right side of Figure 8: the function-call chain need not be invoked sequentially from $F_1$ to $F_6$, leading to high latency. Instead, the individual workers can re-invoke those function calls locally and concurrently. As a result, all functions can execute in parallel and save on latency and network overhead ($t_4 - t_1$ in Figure 8). Furthermore, caching does not require user input, is transparent to the API, and is independent of the synchronous or asynchronous specification. Nonetheless, synchronous calls can be automatically transformed into asynchronous ones under certain conditions [5, 51].

**Conditions for Parallel Function Re-invocation** Intuitively, if the parameters with which, e.g., $F_2$ is called, and the RW set of $F_2$ remains the same, we can safely assume that function $F_2$ can be invoked concurrently without having to be invoked sequentially by $F_1$. If those functions are successfully completed and they acknowledge their completion to the root function $F_1$, then the transaction can be committed. To the contrary, if the RW set of any of the functions $F_1 - F_6$ changes, or the parameters of any of the functions along the call chains change, the transaction must be fully re-executed. In that case, Styx will need to reschedule the transaction for the next epoch.

### Early Commit Replies via Determinism

Implementing Styx as a fully deterministic dataflow system offers several advantages, including the ability to communicate transaction commits to external systems (e.g., clients) before state snapshots are persisted to durable storage. A traditional transactional system can respond to the client only when *i*) the requested transaction has been committed to a persistent, durable state or *ii*) the write-ahead log is flushed and replicated. In Styx’s case, this would occur when an asynchronous snapshot completes (i.e., is persisted to durable storage such as S3), resulting in high latency.

Since Styx implements a deterministic transactional protocol executing an agreed-upon sequence of transactions among the workers, after a failure, the system would run the same transactions with exactly the same effects. This determinism enables Styx to give early commit replies: *the client can receive the reply even before a persistent snapshot is stored.* The assumption here is that the input queue, which persists client requests, will provide Styx’s sequencers with the requests in the same order after replay, a guarantee typically provided by most modern message brokers. Performing state mutations and message passing before persistence has also been explored in DARQ’s speculative execution [42].

## Fault Tolerance

Styx implements a coarse-grained fault tolerance mechanism. Instead of logging each function execution, it adopts a variant of existing checkpointing mechanisms used in streaming dataflow systems [7, 9, 55]. Styx asynchronously snapshots state and stores it in a replicated fault-tolerant blob store (e.g., Minio / S3), enabling low-latency function execution. We describe Styx’s fault tolerance mechanism below.

**Algorithm 1:**
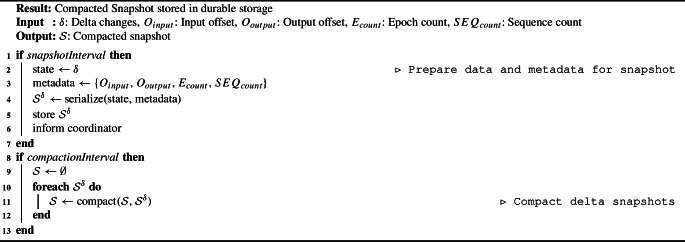
Snapshotting Mechanism

**Algorithm 2:**
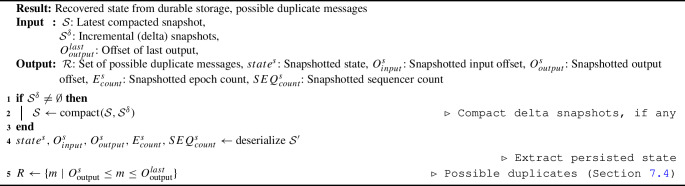
Recovery Mechanism

### Incremental Snapshots & Recovery

The snapshotting mechanism of Styx resembles the approach of many streaming systems [4, 7, 25, 32], that extend the seminal Chandy-Lamport snapshots [9]. Modern stream processing systems checkpoint their state by receiving snapshot barriers at regular time intervals (epochs) decided by the coordinator. In contrast, Styx leverages an important observation: workers do not need to wait for a barrier to enter the system to take a snapshot, since the natural barrier in a transactional epoch-based system like Styx occurs at the end of a transaction epoch.

**Snapshotting** To this end, instead of taking snapshots periodically by propagating markers across the system’s operators, Styx aligns snapshots with the completion of transaction epochs to take a consistent cut of the system’s distributed state, including the state of the latest committed transactions, the offsets of the message broker, and the sequencer counters (*lc*). The minimal information included in the snapshot is $O(N + c)$, where *N* is the number of updates affecting the delta map, and *c* is the fixed number of integers stored for the Kafka offsets and the sequencer variables.

When the snapshot interval triggers, Styx copies the current state changes to a parallel thread and asynchronously persists incremental snapshots, allowing Styx to continue processing incoming transactions while the snapshot operation runs in the background. The snapshotting process is described in Algorithm 1.

**Recovery** In case of a system failure, Styx *i*) rolls back to the epoch of the latest completed snapshot, *ii*) loads the snapshotted state, *iii*) rolls back the replayable source’s topic partitions (that are aligned with the Styx operator partitions) to the offsets at the time of the snapshot, *iv*) loads the sequencer counters, and finally, *v*) verifies that the cluster is healthy before executing a new epoch. The recovery process is described in Algorithm 2.

**Fig. 9:**
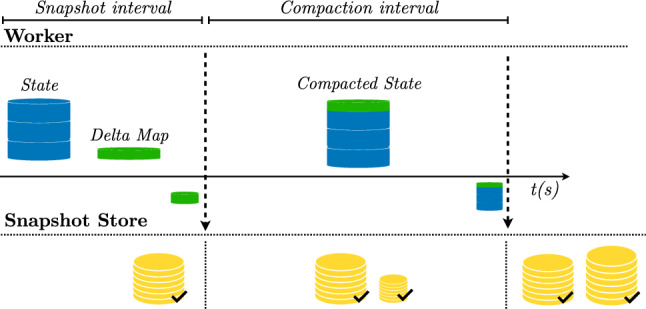
Incremental snapshots with Delta Maps in Styx

**Incremental Snapshots & Compaction** Each snapshot stores a collection of state changes in the form of *delta maps*. A delta map is a hash table that tracks changes to a worker’s state within a given snapshot interval. When a snapshot is taken, only the delta map containing the state changes of the current interval is snapshotted. To avoid tracking changes across delta maps, Styx periodically performs compactions where the deltas are merged in the background, as shown in Figure 9. The cost of compacting is equivalent to the cost of merging two hashmaps with the same key-spaces (*O*(*N*)). The total cost will be $O(M*N)$, with *M* denoting the number of deltamaps to be compacted.

### Sequencer Recovery

To guarantee determinism, upon recovery, Styx’s sequencer must generate sequences identical to those generated between the latest snapshot and the failure. The recovery protocol of the sequencer operates as follows: First, during the snapshot, we store the local counter of each sequencer partition (*lc*), its ID (*sid*), and the epoch counter. Additionally, at the start of each epoch, Styx logs the number of transactions in that epoch, denoted as the epoch size. Logging epoch sizes is necessary due to Styx’s varying epoch sizes and the sequencer rebalancing scheme (Section 5.1). After failure, the recovered sequencer partitions are initialized with the snapshot’s *lc* and *sid*. Afterward, each partition retrieves from its log all the sizes of all epochs executed since the last snapshot. Finally, after recovery, the sequencer matches the epoch sizes to those recorded in the log, yielding the same global sequence as before failure.

### Exactly-Once Processing

Initially, the durable input queue, which serves as a replayable source, enables Styx to replay requests after failures. By rolling back the queue partitions (aligned with Styx’s operator partitions) to their respective offsets recorded in the latest snapshot, Styx can reprocess only transactions whose state changes are not yet reflected in the snapshot. Transactions committed and early-commit replies stored in the egress can be deduplicated (Section 7.4).

Styx executes each transaction to completion within a single epoch. A given transaction can execute a large call-graph of functions that can affect the state. If a failure occurs, the transaction’s state effects are restored to the latest snapshot, and the entire transaction is re-executed. As a result, no special attention is required to ensure that remote function calls are executed exactly-once, except for resetting all TCP channels between Styx’s workers after recovery.

#### Lemma 1

The state mutations of committed transactions in Styx are reflected exactly-once, even upon failure.

#### Proof

Let $S_t$ denote the state of the system at time *t*. $Q_t = \{r_1, \dots , r_n\}$ denotes the durable input queue at time *t* that holds all requests $r_i$ to be processed. We assume that the input queue operates as FIFO and requests $r_i$ are deterministic. Each $r_i$ will be sequenced as a transaction $T_i = \{upd_l, func_m\}$ by a deterministic sequencer, where $upd_l$ are the state updates and $func_m$ are the function calls of the transaction. We assume that $upd_l$ occurs atomically and that $func_m$ is also reflected once, given the use of a reliable communication protocol. Given the same initial state *S* and input from *Q*, it always produces the same state transition $S \rightarrow S'$,which means $S'_{t+1} = mutation(S_t, Q_t)$. The execution of a transaction $T_i$ is deterministic.

At any time *t*, the state of the system $S_t$ reflects all transactions in $Q_t$ that have been fully executed and committed. Accordingly, the state $S_t$ ignores partially executed or in-progress transactions in $Q_t$. We denote the latest durable snapshot taken up to time *t*, as $\text {Snapshot}(S_t, i, n)$ where *n* corresponds to the offsets of the first request $r_i$, and last request $r_n$ of the input queue to be processed up to time *t*. Upon failure, a subset of $Q_t$, $Q^{success}_t = \{r_1, \dots , r_k\}$ will contain successfully committed transactions and a subset $Q^{fail}_t = \{r_{k+1}, \dots , r_n\}$ will contain aborted transactions such that $Q_t = Q^{success}_t + Q^{fail}_t$. To recover from a failure, $Q_t$ is rolled back to $S_t$ from $\text {Snapshot}(S_t, i, n)$ as we persist the offsets of our input queue. Transactions in $Q_t$ are replayed in the original order from offset *i* to offset *n* of our input queue. This is ensured by the FIFO queue and the deterministic sequencer. After processing the input transactions, $Q^{success}_t$ includes requests already reflected in $\text {Snapshot}(S_t)$, and $Q^{fail}_t$ includes pending requests. Since $\text {Snapshot}(S_t)$ reflects $Q^{success}_t$ and $Q_t = Q^{success}_t + Q^{fail}_t$, the replay and processing ensure: $S_{t+1}'' = mutation(S_t, Q^{fail}_t) = S_{t+1}'$. Thus, the effects of all transactions will be reflected in the state exactly-once, even after failure. $\square$

### Exactly-Once Output

A common challenge in fault tolerance for streaming systems is achieving exactly-once output [19, 22] in the presence of failures, which is difficult to achieve for low-latency use cases. For example, in Apache Flink’s [8] exactly-once output configuration, clients can only retrieve responses after they have been persisted in a snapshot or a transactional sink. This arrangement is sufficient for streaming analytics but not for low-latency transactional workloads, as discussed previously in Subsection 6.4.

To solve that, during recovery, Styx: *i*) reads the last offset of the egress topic, *ii*) compares it with the output offset persisted in the snapshot, determining for which transactions the clients have already received replies, *iii*) retrieves the TIDs attached in those replies, and *iv*) does not send a reply again to the egress topic for those transactions. Note that this deduplication strategy is based on the assumption that TIDs are assigned deterministically.

**Fig. 10:**
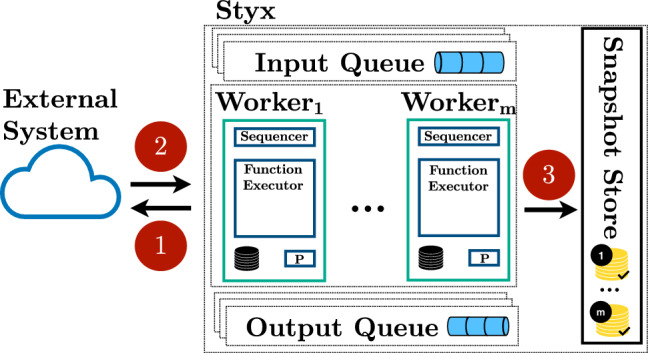
External system call critical points and Styx

### Addressing Non-Deterministic Functions

As discussed in Section 7.1, Styx’s recovery mechanism is based on deterministic replay. To this end, Styx requires that the functions authored by developers are also deterministic: replaying the same function multiple times with the same inputs and database state should yield the same results. However, one can achieve determinism even in the presence of non-deterministic logic inside functions, such as randomness (e.g., random numbers/sampling) or calls to external systems (e.g., calling an external database or API). Styx can follow the approach of existing systems (e.g., Temporal, Clonos [55]). In the following, we explain how this can be achieved.

**Randomness** To retain determinism in the case of randomness, Styx can use an external fault tolerant write-ahead log (WAL) to log the random number along with the TID. Thus, in the case of failure and replay, Styx can use the logged random number, essentially making the function call deterministic during replay.

**Calls to External Systems** As illustrated in Figure 10, an interaction with an external system needs to consider three critical points to maintain determinism. Styx assumes that the external system supports idempotency, meaning that if a call is made twice with the same idempotency key, the effects on the external system’s state and its return value will remain the same. In , Styx must log the idempotency key and the TID to the WAL before calling the external system. If the external system produces a response (), Styx can store it in the WAL and retrieve it from there in case of replay. Finally, when Styx completes a snapshot (), it can also clear the WAL for garbage collection, as the prior entries are no longer needed.

Finally, Styx could mask those operations behind an API that exposes the following functionality, such as system_x.random for generating random numbers and system_x.call_external for making external system calls.

## State Migration

Implementing transactions on top of dataflows adds additional challenges to state migration support in Styx. Methods that strictly target SPEs for state migration  [16, 27, 31] do not apply to our use case since they do not support transactional semantics. On the other hand, state migration methods for transactional databases [3, 18, 53] are tightly coupled to the traditional OLTP database architecture and cannot be directly applied to a dataflow engine such as Styx. Therefore, Styx requires a new, tailor-made approach to maintain its transactional and exactly-once execution guarantees, while also adapting to its snapshotting mechanism.

The most straightforward approach to migrating state in any system is the Stop-and-Restart (S&R) method, in which the system halts processing of incoming requests, reassigns the data, and then restarts processing. More sophisticated approaches adopt some of the following mechanisms to migrate state: *i*) maintain state replicas across workers to minimize the amount of data in need of migration [3, 16, 31], *ii*) on-demand migration that only sends the data once a worker requires it and [18, 27], *iii*) async migration to transfer data during idle time to progress a migration asynchronously [3, 18].

In Styx, we implement two state migration approaches: a baseline S&R version tailored to Styx, in line with approaches that pause and restart execution (e.g., Flink Rescaling), and an approach denoted as Online Migration (OM), which combines elements of migration approaches (*ii*) and (*iii*), in line with  [16, 27, 31]. In this section, we first present an overview of state migration in Styx, specify the migration stages (triggering, handling, and resumption of processing), and describe the changes we made to Styx to support these stages. We then elaborate on the S&R and OM methods and discuss how we maintain fault tolerance, determinism, end-to-end exactly-once semantics, and serializability during state migration. In this work, we aim to extend Styx by introducing elasticity, the first step toward making Styx serverless.

### State Migration Overview

To support transactional state migration, we extend Styx’s base core runtime. This section outlines the architectural modifications required to enable this capability.

We assume that migration is initiated by an external client or Styx’s coordinator based on metrics such as load imbalance or resource utilization. The migration-triggering policy, including autoscaling heuristics and monitoring, is considered orthogonal and left for future work. To enable correct and efficient state movement, Styx introduces the following system-level changes:

**Partition-level State and Metadata** To make state movement more flexible, operator state and Kafka offsets are tracked at a finer granularity; specifically, on a per-partition basis. Previously, state, movement, and offsets were maintained per operator, which prevented distinguishing among multiple partitions of the same operator on a single worker. The finer-grained tracking enables selective state migration without relying on hashing to determine a key’s partition or requiring complete operator-level checkpoints.

**Shadow Partitions** Styx’s internal reconfiguration is not guaranteed to be aligned with the partitions of messages coming from the input queue (Kafka). To address this potential misalignment, Styx temporarily uses shadow partitions to forward any out-of-place transactions to the correct partitions, which are responsible for the relevant keys after reconfiguration. In that way, Styx ensures correctness without requiring global input suspension. This issue is particularly evident during downscaling, when partitions are removed. For instance, when a client initiates a downscale migration by triggering reconfiguration (repartition), other clients may continue sending transactions to Styx under the previous partitioning scheme. In this case, transactions must access partitions that no longer exist. Keeping old partitions as shadow partitions, which only forward transaction requests to the correct partitions without any state mutation responsibilities, is essential for Styx to preserve exactly-once processing guarantees.

**Global Offset Restoration** A rerouting mechanism is required not only during downscaling involving shadow partitions but also as part of the general migration solution. Its role is twofold: *i*) detect incoming transactions from the input queue that are routed to an outdated partition due to client partitioning misalignment, and *ii*) reroute them to the correct partition. Following our previous example, a transaction may be routed to an outdated partition until all clients of Styx have updated their routing tables to align with the new partitioning scheme. To maintain exactly-once processing and output, it is essential to restore the input/output queue offsets, as they may be updated in at most two places (the previous and new partitioning). This ensures that no records are skipped or reprocessed during migration in case of failure.

**Blocking Actions Minimization** To ensure low latency even in the presence of large data transfers during migration, we had to make the two following adjustments to Styx *i*) add compression to large messages and, *ii*) stream asynchronous snapshots. First, Styx enforces compression using the Zstandard algorithm [13] for messages larger than a configurable threshold (by default 4KB). Second, regarding the snapshot mechanism in Styx, Styx now spawns a background thread that receives state deltas in a streaming fashion to prevent blocking when a delta becomes large (i.e., under heavy load, the subset of keys involved is significantly large). In the previous version, Styx accumulated the entire delta and then sent it to the background thread, resulting in significant latency spikes that are now resolved.

**Composite Key Partitioning** In its current version, Styx also supports composite key partitioning to enhance data locality. Keys can be grouped by logical attributes (e.g., *warehouse_id* in TPC-C, which is a prefix in all table primary keys except the *item* key), allowing transactions to access co-located partitions. During migration, Styx utilizes this structure to co-locate groups of related keys with the same worker. This optimization reduces cross-worker communication and improves transaction commit latency.

These design extensions enable Styx to support both synchronous (stop-and-restart) and asynchronous (online) migration strategies without compromising transactional or fault tolerance guarantees, while maintaining low latency during reconfiguration.

**Algorithm 3:**
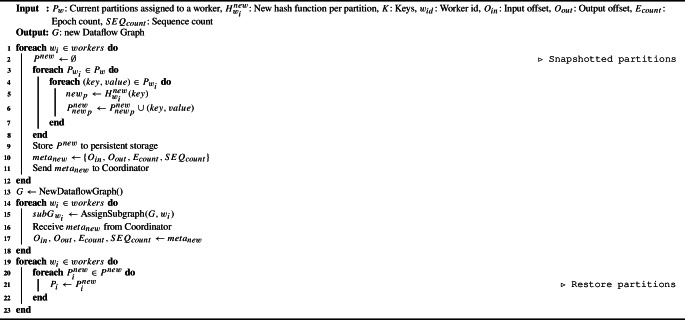
Stop and Restart

**Fig. 11:**
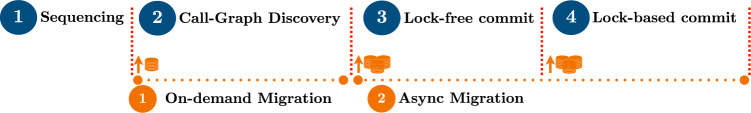
Alignment of epoch phases with online state migration. In phase  transactions determine their call-graphs, and therefore, the associated keys need to be migrated immediately. By contrast, in  and , keys can be migrated asynchronously without interfering with the transactional protocol

**Fig. 12:**
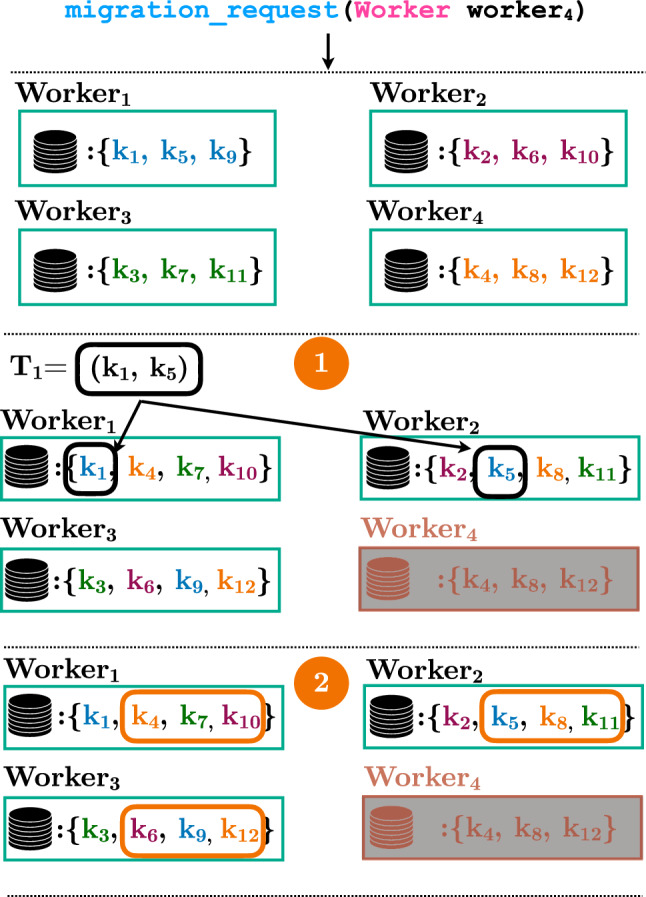
State distribution before and after migration request, which removes $worker_4$. After migration, we observe a transaction $T_1 = \bigl \{k_1, k_5\bigr \}$. $T_1$ interacts with keys $k_1$ and $k_5$, meaning that while $k_1$ is already in-place, $k_5$ needs to be migrated on-demand as migration phase  suggests in Figure 11. The rest of the keys, assuming that there is no other transaction interacting with them, can be migrated asynchronously in the migration phase

### Stop & Restart

Stop-and-Restart (S&R) is the most straightforward migration strategy that we could implement on top of Styx. It suspends execution, performs state migration, and resumes computation with updated routing. Styx implements an optimal variant of S&R tailored to its transactional runtime. In Algorithm 3, we detail S&R where, at first, for each worker ($w_i$) and all the partitions assigned to them ($P_{w_i}$) Styx rehashes all the keys of that partition based on the new partitioning using its hash function ($H^{new}_{w_i}$) and adds them alongside their values to the new partitions ($P_{new}$). Once a worker completes the hashing step, it stores the new partitions as a snapshot in persistent storage. Then, each worker sends their metadata (input/output offsets, sequencer count, and epoch count) to the coordinator, concluding the ‘Stop’ step. To ‘Restart’ Styx based on the new partitioning, each worker is assigned its new partitions from the graph and receives the updated metadata from the coordinator. Finally, the worker loads the new partitions from the previously rehashed snapshot stored in persistent storage.

Although simple, robust, and independent of the transactional protocol, S&R incurs downtime due to rehashing, data storage, and data loading. This process violates availability, making S&R more suitable for planned migration settings rather than Styx’s serverless requirements.

**Algorithm 4:**
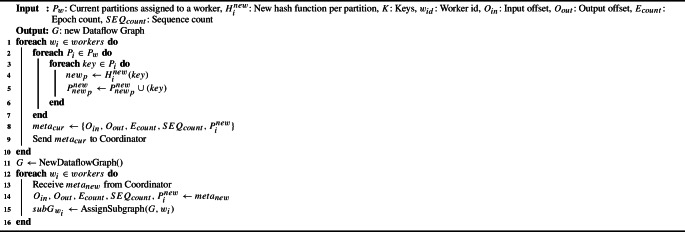
Online Migration

### Online Migration

To support online, near-zero-downtime migration, Styx introduces an online method adapted to its transactional model. In contrast to S&R, the online method performs migration on demand and asynchronously, allowing the system to remain available throughout. Styx uses its transactional epoch protocol to integrate migration steps into regular processing cycles. As shown in Figure 11, keys accessed during Phase  are migrated synchronously and on-demand to ensure consistency. Other keys are migrated asynchronously in phases  and  during idle times, utilizing batching mechanisms. Note that, if the transaction’s read-write set changes during , the transaction will abort and be rescheduled for the next epoch, as described in 6.2. Thus, keys that are part of such a transaction’s read-write set will be processed either asynchronously or by next epoch’s on-demand migration phases.This asynchronous migration is essential for the migration to complete (i.e., a key might not be accessed at a reasonable time from any transaction), which is necessary for the fault tolerance mechanism to be reactivated (snapshots are switched off mid-migration as per the SotA approaches to maintain consistent snapshots). While epoch phases are used to initiate async/sync migration actions, async key migration is not constrained by the epoch-ending barrier. When a batch of keys is selected for async migration, the change is atomically registered in the worker. If an on-demand migration request arrives for keys that are already in-flight, the source worker notifies the requester that the keys are already scheduled for delivery, ensuring consistency. However, async migration batch sizes are typically tuned to complete before the next on-demand requests phase. Styx avoids blocking the async migration at the epoch boundary because synchronizing these processes would increase epoch duration and reduce throughput.

In Algorithm 4, we detail the Online Migration method. More precisely, for all partitions currently assigned to a worker ($P_{w_i}$), Styx first rehashes every key contained in the partition based on the new partitioning scheme using its hash function ($H^{new}_i$). Differentiating from Stop-and-Restart (S&R), Styx does not directly move or copy the key-value pairs. Instead, it creates a routing table ($P^{new}$) that indicates the destination partition ($P^{new}_{new\_p}$), for every key. Once a worker finishes this hashing and routing table creation step, it packages this routing information alongside its critical metadata, including the current input and output offsets ($O_{in}$, $O_{out}$), sequencer count ($SEQ_{count}$), and epoch count ($E_{count}$), and sends this entire package ($meta_{cur}$) to the coordinator. The coordinator then constructs the new dataflow graph (*G*). Finally, the worker receives the finalized new metadata ($meta_{new}$) and routing tables from the coordinator, loads them, and proceeds to perform the actual state migration concurrently with the transactional protocol using the on-demand () and asynchronous () mechanisms, as illustrated in Figures 11 and 12.

Figure 12 illustrates a down-scaling action with repartitioning, moving from 4 to 3 workers, to demonstrate how the on-demand and asynchronous state migration processes are executed. Figure 12 includes two critical points: *i*) how the state is distributed before and immediately after the migration request, which removes $worker_4$, and *ii*) how transactions can directly request keys; this action directly triggers their migration (on-demand). For example, a transaction, $T_1 = \bigl \{k_1, k_5\bigr \}$, which starts executing in the new configuration, interacts with keys $k_1$ and $k_5$. Since $k_1$ is already located in its post-rehash destination, it is in-place. However, $k_5$ is found in its old location and needs to be migrated on-demand before $T_1$ can complete, corresponding to the migration phase  suggested in Figure 11. The rest of the keys originating from the old partitions, assuming no other active transaction accesses them, are migrated asynchronously in the background, corresponding to migration phase .

### Maintaining Guarantees During Migration

Both state migration approaches need to preserve Styx’s correctness guarantees, namely: *i*) exactly-once processing, *ii*) determinism, *iii*) serializable transactional guarantees and, *iv*) exactly-once output. For the S&R method, maintaining correctness is straightforward since it stops execution, shuffles the data, and restarts. It does not affect any of the already-in-place mechanisms of Styx detailed in Section 7. Thus, in this subsection, we will primarily explain how the Online Migration method’s operation satisfies Styx’s correctness guarantees.

Styx maintains deterministic execution and serializability guarantees throughout Online Migration. When a transaction requires access to a key located in another worker (triggering On-Demand Migration), the worker blocks execution until that key is received. This procedure is safe because Styx’s single-process coroutine approach ensures that no other transaction on the same worker can simultaneously request the same key. Transactions can operate only on fully available, up-to-date keys, and migrations are aligned with epochs to ensure consistency. Additionally, the asynchronous phase of Online Migration is performed only after the call-graph of all transactions within the epoch has been discovered. At that point, all the requested key transfers of the on-demand migration phase are guaranteed to have been completed. Moreover, fault tolerance remains unaffected; if a failure occurs, Styx will recover from the latest snapshot and restart the migration without compromising correctness. For the same reason, exactly-once processing and output remain unaffected by the migration mechanism.

Finally, the only critical point to be addressed in both the S&R and Online migration methods is out-of-partition events due to client-server partitioning misalignment. In Section 8.1, we explained the two new mechanisms of Styx that address this issue, namely Shadow Partitions and global offset restoration. Shadow partitions are used temporarily to reroute out-of-partition transactions from Kafka, ensuring that the correct worker and partition process the incoming transaction. To fully address this issue, the Kafka offset progress that may be affected by two different workers is restored by the coordinator before it is stored in a snapshot. This coordination ensures exactly-once processing in the case of failure during state migration.

## Evaluation

We evaluate Styx by answering the following questions:

- Section (9.2) How does Styx compare to State-of-the-Art serializable transactional SFaaS systems?
- Section (9.2) How does Styx perform under skewed workload?
- Section (9.3) How well does Styx scale?
- Section (9.4) Does the snapshotting mechanism affect performance?
- Section (9.5) How well do different state migration mechanisms work in Styx?

### Setup

**Systems Under Test** In the evaluation, we include SFaaS systems that provide serializable transactional guarantees. Those are:

*Beldi *[64]*/Boki *[33] Both systems use a variant of two-phase commit and Nightcore [34] as their function runtime and store their data in DynamoDB. Additionally, Boki is deployed with the latest improvements of Halfmoon [52].

*T-Statefun *[29] T-Statefun maintains the state and the coordination of the two-phase commit protocol within an Apache Flink cluster and ships the relevant state to remote stateless functions for execution. For fault tolerance, it relies on a RocksDB state backend that performs incremental snapshots.

*Styx* Styx is implemented in Python 3.13, contains the optimizations mentioned in Section 8.1 and uses coroutines to enable asynchronous concurrent execution. Apache Kafka is used as an ingress/egress, and Minio/S3 as a remote persistent store for Styx’s incremental snapshots. Finally, Styx is a standalone containerized system that works on top of Docker and Kubernetes for ease of deployment.

**Workloads/Benchmarks** Table 1 summarizes the three workloads used in the experiments.

*YSCB-T *[17] We use a variant of YCSB-T [17], in which each transaction consists of two reads and two writes. The concrete scenario is as follows: First, we create 10.000 bank accounts (keys) and perform transactions in which a debtor attempts to transfer credit to a creditor. This transfer is subject to a check of the debtor’s creditworthiness to ensure the payment can be fulfilled. If not, a rollback is required. The selection of a relatively small number of keys is deliberate: we want to assess the system’s ability to sustain transactions under high contention. In addition, for the experiment depicted in Figure 14 (skewed distribution), we select the debtor key based on a uniform distribution and the creditor based on a Zipfian distribution, where we can vary the level of contention by modifying the Zipfian coefficient.

*Deathstar *[24] We employ Deathstar [24], as adapted to SFaaS workloads by the authors of Beldi [64]. It consists of two workloads: *i*) the Movie workload implements a movie-review service in which users write reviews of movies. *ii*) the Travel workload implements a travel reservation service where users search for hotels and flights, sort them by price/distance/rate, find recommendations, and transactionally reserve hotel rooms and flights. Both Deathstar workloads follow a uniform distribution. Note that T-Statefun could not be used in these experiments because it does not support range queries.

*TPC-C *[41] The prime transactional benchmark targeting OLTP systems is TPC-C [41]. In our evaluation, we employ the NewOrder and Payment transactions, and we had to rewrite them into the SFaaS paradigm, splitting the NewOrder transaction into 20-50 function calls (one call for each item in the NewOrder transaction) and the Payment transaction into 8 function calls. TPC-C scales in size/partitions by increasing the number of warehouses represented in the benchmark. While a single warehouse results in a skewed workload (all transactions are directed to the same warehouse), increasing the number of warehouses reduces contention, thereby allowing higher throughput and lower latency. Note that the TPC-C experiments do not include Beldi, Boki, or T-Statefun because they are not supported.

**Table 1:** Workload characteristics.

Scenario	#keys	Function Calls	Transactions %
**YCSB-T**	10k	2	100%
**Deathstar Movie**	2k	9-10	0%
**Deathstar Travel**	2k	3	0.5%
**TPC-C**	1m-100m	8 / 20-50	100%

*State Migration Experiments* The workloads used in our state migration experiments follow the SotA transactional approaches [3, 18], which are the YCSB [14] and TPC-C benchmarks. We use two YCSB datasets: a smaller dataset with 1 million records (1GB) for small-state experiments and a larger one with 10 million records (10GB). Each record consists of a primary key and 10 columns containing 100-byte randomly generated strings. We follow the workload configuration from [18], which includes two transaction types: 15% of operations perform a single-record update, while the remaining 85% perform a single-record read. It is important to note that YCSB differs from the YCSB-T variant mentioned above. In YCSB-T, keys and values are single integers, which lead to small state sizes and limits the evaluation of state migration scenarios. For TPC-C, we reuse the setup described in Section 9.1 and generate two datasets, a small one (10 warehouses, 1GB) and a larger one (100 warehouses, 10GB). All tables in both benchmarks are partitioned into 16 parts.

**Resources** For Beldi/Boki, T-Statefun, and Styx, we allocated a total of 112 CPUs, each with 2 GB of RAM, matching the configuration presented in the original Boki paper [33]. Additionally, across all evaluation scenarios, the data fit within memory on all systems. Unless stated otherwise, Styx and T-Statefun are configured to perform incremental snapshots every 10 seconds. All external systems, i.e., DynamoDB (Beldi, Boki), Minio, and Kafka (Styx, T-Statefun), are configured with three replicas for fault tolerance.

*External Systems* Boki and Beldi use a fully managed AWS DynamoDB instance, which does not specify the number of resources it consumes and is in addition to the 112 CPUs assigned to Boki and Beldi. Similarly, the resources assigned to Minio/S3 (Styx and T-Statefun) are not accounted for.

**Metrics** Our goal is to observe systems’ behavior, measured by their latency while varying the input throughput.

*Input/Output throughput* represents the number of transactions submitted per second to the system under test. As input throughput increases during an experiment, we expect the latency of individual transactions to rise until aborts begin to occur due to contention or high load. In the migration experiments, we also display the output throughput, which is the number of transaction responses Styx produces per second. During migration, we expect *i*) the input throughput to remain stable, since we do not pause the clients, and *ii*) the output throughput to decrease.

*Latency* represents the time interval between submitting a transaction and the reported time when the transaction is committed/aborted. In Styx and T-Statefun, the latency timer starts when a transaction is submitted in the input queue (Kafka) and stops when the system reports the transaction as committed/aborted in the output queue. Similarly, in Beldi and Boki, latency is the time between when the input gateway receives a transaction and when the gateway reports that the transaction has been committed/aborted. In all latency experiments, we report the median latency (50p) and tail latency (99p).

*Migration Interval* An important migration-only metric is the migration interval, or how long the migration process takes. To demonstrate this, we plot the start and end migration timestamps across all state migration experiments.

### Latency vs. Throughput

**Fig. 13:**
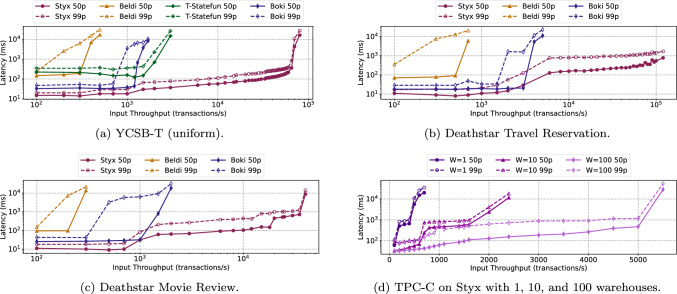
Evaluation in different scenarios. T-Statefun does not support range queries required by the Deathstar workloads. TPC-C is only supported by Styx

We first study the latency-throughput tradeoff of all systems. We keep the number of resources allocated to the systems constant (112 CPUs) while progressively increasing the input throughput. We measure the transaction latency. As depicted in Figure 13, Styx outperforms its baseline systems by at least an order of magnitude. Specifically, in YCSB-T Figure (13a), Styx achieves a performance improvement of ~20x in terms of throughput against T-Statefun, which ranks second. In addition, Styx outperforms Boki by ~30x in Deathstar’s travel reservation workload Figure (13b) and by ~35x in Deathstar’s movie review Figure 13c) workload. Finally, in the TPC-C benchmark Figure (13d), which requires a large number of function calls per transaction (20-50), we observe that Styx’s performance improves as we increase the input throughput for different numbers of warehouses, reaching up to 3K TPS with sub-second 99$^{\text {th}}$ percentile latency (100 warehouses).

**Fig. 14:**
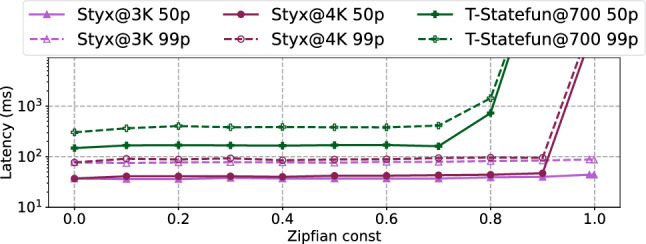
Latency evaluation for varying levels of contention (0.0 - 0.999) with YCSB-T (skewed). We ran Styx with two input-throughput variations to clearly demonstrate its behavior under contention. Note that Styx and T-Statefun execute all transactions to completion (abort%=0)

**Aborts & Throughput** Beldi and Boki follow a no-wait-die concurrency control approach, which leads to a significant amount of aborts as the throughput increases. Styx and T-Statefun do not use such a transaction abort mechanism. Instead, they execute all transactions to completion. This difference in transaction handling under high load makes latency comparisons across systems difficult. For this reason, in Figure 14, we plot the results of Styx and T-Statefun and present the performance of Beldi and Boki in a separate table (Figure 2), alongside their abort rates.

**Table 2:** Evaluation of Boki and Beldi for varying levels of contention with YCSB-T. We report the abort ratio and the committed transaction rate, and omit latency because the systems do not execute all transactions to completion. Both run at their maximum sustainable throughput.

		0.0	0.2	0.4	0.6	0.8	0.9	0.99	0.999
Beldi	Abort %	47.93	45.54	44.31	47.28	52.40	56.06	61.62	60.70
	CMT TPS	104	108	111	105	95	76	76	78
Boki	Abort %	48.77	48.23	49.54	51.82	61.29	68.50	74.47	70.71
	CMT TPS	359	362	353	337	271	220	179	205

We observe the following: *i*) at the highest level of contention (*Zipfian* at 0.999) Styx achieves at least 2000 TPS, outperforming the rest by ~5-10x in terms of effective throughput, *ii*) both Beldi and Boki (that run at their maximum sustainable throughput) abort more transactions as the level of contention increases (~40-70%), which significantly impacts their effectiveness as shown in Table 2, and *iii*) Styx shows an increase in latency only in high levels of contention ($Zipfian>0.99$) while executing at ~4x higher throughput than the rest.

**Fig. 15:**
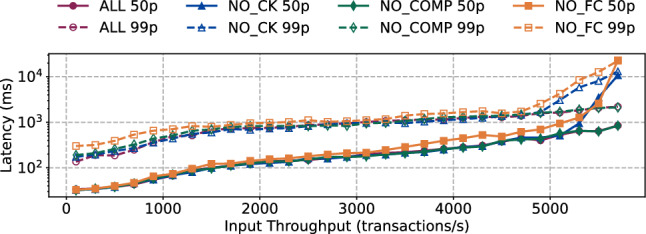
Ablation study of Styx optimizations. We report p50 and p99 latency for TPC-C with 100 warehouses under different optimization configurations

**Optimizations’ Impact** We evaluate the impact of individual optimizations on the transactional execution path of Styx by rerunning TPC-C with 100 warehouses under four configurations: all optimizations enabled, without composite keys (NO_CK), without compression (NO_COMP), and without the fallback cache (NO_FC). Figure 15 reports p50 and p99 latencies across input throughput.

Overall, the optimizations have little impact on median latency, indicating that steady-state transactional performance is primarily dominated by the core transactional protocol. The fallback cache is the main exception, reducing p50 latency by approximately 15-25% beyond 3,000 TPS, p99 latency by 5-25% in all throughputs, and significantly stabilizing latency near saturation. Disabling composite keys primarily affects p99 latency under high load, whereas compression has a negligible impact in this OLTP-heavy setting because messages remain relatively small.

**Takeaway** These results show that Styx’s performance gains are not driven by auxiliary engineering optimizations, but stem from a transactional core that remains efficient and robust, with optimizations mainly improving tail behavior and system stability under high load.

**Runtime Breakdown** In Table 3, we show where the systems under test spend their processing time. We use YCSB-T for this purpose since it is the only benchmark supported by all the systems (Section 9.1). We measured the median latency while all systems were operating at 100 TPS for 60 seconds, and averaged the proportions of function execution, networking, and state access across all committed transactions. The key observations are: *i*) Styx’s co-location of processing and state resulted in minimal state access latency, and *ii*) Styx’s asynchronous networking enabled lower network latency.

**Takeaway** The performance advantages of Styx across all experiments are enabled by the following three properties and design choices: *i*) the co-location of processing and state with efficient networking as shown in Figure 3, contrary to the other systems that have to transfer the state to their function execution engines; *ii*) the asynchronous snapshots with delta maps for fault tolerance compared to the replication of Beldi/Boki and the LSM-tree-based incremental snapshots of T-Statefun; *iii*) the efficient transaction execution protocol employed in Styx compared to the two-phase commit used by Styx’s competition.

**Table 3:** Performance breakdown of all systems. (median latency - percentage from the total)

System	Function Execution	Networking	State Access
**Styx**	**0.31ms** - 2.2%	**13.43ms** - 95.6%	**0.30ms** - 2.2%
**Boki**	1.1ms - 3.3%	16.1ms - 49%	15.68ms - 47.7%
**T-Statefun**	2.76ms - 2.2%	92.12ms - 74.3%	29.11ms - 23.5%
**Beldi**	1.01ms - 0.7%	56.58ms - 38.4%	89.57ms - 60.9%

**Fig. 16:**
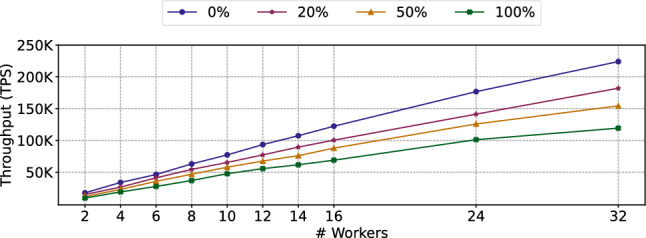
Scalability of Styx on YCSB-T with varying percentages of multi-partition transactions

**Fig. 17:**
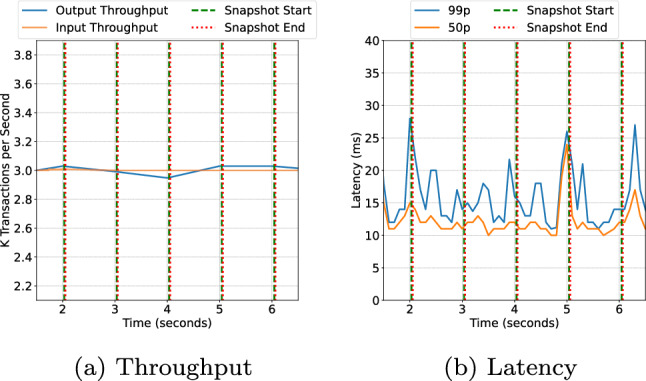
Impact of Styx’s snapshotting on performance

**Fig. 18:**
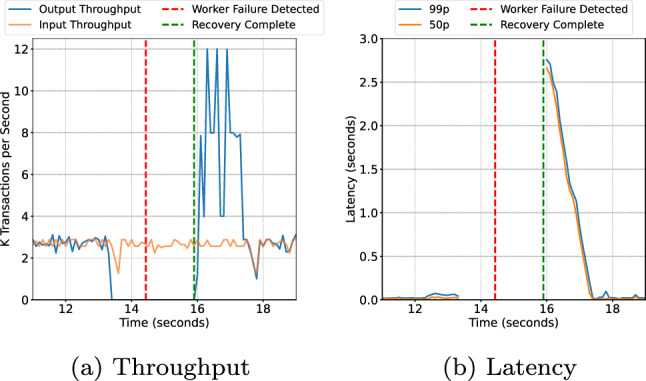
Styx’s behavior during recovery

### Scalability

In this experiment, we test the scalability of Styx by increasing the number of Styx workers. Each worker is assigned 1 CPU and a state of 1 million keys. We measure the maximum throughput on YCSB-T. The goal is to calculate the speedup of operations as the input throughput and number of workers scale together. In addition, we control the percentage of multi-partition transactions in the workload, i.e., transactions that span across workers. In Figure 16, we observe that in all settings, Styx retains near-linear scalability. Finally, Styx displays the expected behavior as the number of multi-partition transactions increases.

### Fault-Tolerance Evaluation

**Effect of Snapshots** In Figure 17, we depict the impact of the asynchronous incremental snapshots to Styx’s performance. In both figures, we indicate the start and end times of each snapshot. The state includes 1 million keys, and we use a 1-second snapshot interval. Styx is deployed with four 1-CPU workers, and the input transaction arrival rate is fixed to 3K YCSB-T TPS. In Figure 17a, we observe that during a snapshot operation, Styx shows virtually no performance degradation in throughput. In Figure 17b, we observe a minor increase in the end-to-end latency in some snapshots. The reason for that is the concurrent snapshotting thread, which competes with the transaction execution thread during snapshotting. At the same time, it must temporarily block the transaction execution thread to copy the corresponding operator’s state delta.

**Fig. 19:**
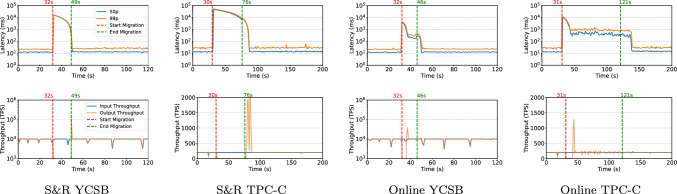
Big State 10 GB Scale Up: Latency (top row) and Throughput (bottom row)

**Fig. 20:**
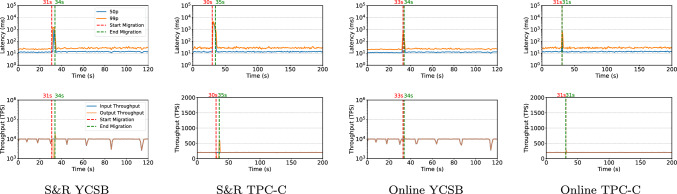
Small State 1GB Scale Up: Latency (top row) and Throughput (bottom row)

**Recovery Time** In Figure 18, we evaluate the recovery process of Styx with the same parameters as in Figure 17. We reboot a Styx worker at ~13.5 seconds. It takes Styx’s coordinator roughly a second to detect the failure. After the reboot, the coordinator re-registers the worker and notifies all workers to load the most recent complete snapshot, merge any uncompacted deltas, and use the message broker offsets for that snapshot. The recovery time is also observed in the latency (Figure 18b) that is ~2.5 seconds (time to detect the failure in addition to the time to complete recovery). In terms of throughput (Figure 18a), we observe Styx working on its maximum throughput after recovery completes to keep up with the backlog and the input throughput.

### State Migration Experiments on Styx

In this section, we evaluate the state migration mechanism of Styx for maintaining sustainable throughput during scaling up and down while repartitioning the entire state. The repartitioning operation entails substantial data movement because Styx’s partitioner is hash-based.

#### State Migration Results

We have run YCSB and TPC-C workloads at scale-up and scale-down with both large and small state sizes.

**Fig. 21:**
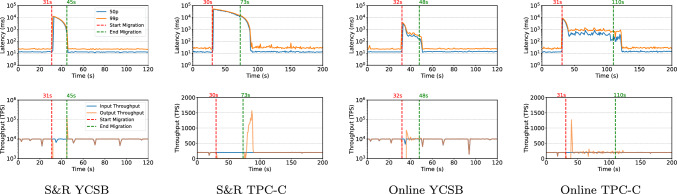
Big State (10GB) Scale Down: Latency (top row) and Throughput (bottom row)

**Scale-Down** In the scale-down scenario, we go from 16 Styx 1-CPU workers down to 12, in addition to repartitioning the state to the same number of partitions. In Figure 21, we observe that the S&R method in both YCSB and TPC-C incurs very high latency (tens of seconds), 14 seconds of downtime in YCSB and 43 seconds of downtime in TPC-C while migrating and repartitioning 10 GB of data. The Online method displays a) a minor latency hike related to the rehash operation at the beginning of the migration phase in both workloads that does not exceed 10 seconds, and b) minimal downtime that is close to 5 seconds in YCSB and 10 seconds in TPC-C. In YCSB, both in small and large state, the migration takes around the same time with a minor advantage compared to the S&R method. By contrast, on TPC-C, the Online method takes twice as long because TPC-C contains more keys that must be transferred, and the async migration is configured to transfer 5,000 keys per transactional epoch.

**Fig. 22:**
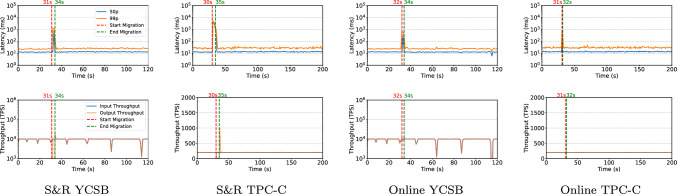
Small State 1GB Scale Down: Latency (top row) and Throughput (bottom row)

In Figure 22 depicting the experiment with the smaller state, we observe the same trends, but the migration impact is much smaller.

**Scale-Up** In the scale-up scenario, we go from 12 Styx 1-CPU workers up to 16, in addition to repartitioning the state to the same number of partitions. In Figures 19 and 20 that display the big and small state scale-up experiments, we observe similar behavior to the scale-down experiments, which is to be expected since both migration methods are agnostic to scale-up/down semantics. The only difference is that migration takes longer since 16 threads perform the initial rehashing phase in the scale-down experiment versus 12 in the scale-up.

**Takeaways** In general, the Online migration method outperforms the S&R method in all the critical performance indicators such as *i*) downtime, where the Online migration is at least 4x faster than stop and restart and *ii*) the peak latency, which does not go above 10 seconds, and once the hashes are computed and Styx catches up to the input, the transactions instantly drop to sub-second latencies. It is important to note that the only metric on which the Online method falls behind is migration time, which can be explained by the fact that the fault-tolerance mechanism is disabled during migration and that the rollback window in the event of failure is larger.

## Related Work

**Dataflow Systems** Support for fault-tolerant execution in the cloud with exactly-once guarantees [7, 20] is one of the main drivers behind the wide adoption of modern dataflow systems. However, they lack a general and developer-friendly programming model that supports transactions and provides a natural way to program function-to-function calls. Closer to the spirit of Styx are Ciel [47] and Noria [26]. Ciel proposes a language and runtime for distributed, fault-tolerant computations that support control flow. Noria addresses the view maintenance problem through a dataflow architecture that quickly propagates updates to clients, targeting web-based, read-heavy computations. However, neither provides a transactional model for workflows of functions such as Styx.

**State Migration** Prior work has explored state migration across transactional and stream processing systems. Squall [18] performs live state migration by locking affected partitions via a dedicated transaction, enabling on-demand data movement but relying on DBMS-level deadlock handling and range partitioning assumptions. Clay [53] incrementally migrates frequently co-accessed keys using a cost model to balance migration overhead and transaction performance. Albatross [15] targets shared-storage systems, incrementally copying in-memory caches and active transaction state while relying on two-phase commit to ensure consistency. Meces [27] enables fine-grained, on-demand state migration in stream processing systems using markers and guarantees exactly-once semantics, but does not consider transactions. Rhino [16] focuses on query reconfiguration in stream processing systems, maintaining state replicas via virtual nodes, but similarly lacks transactional support.

**Autoscaling** Several works address dynamic reconfiguration in SPEs. DS2 [35] is a control-based autoscaler that leverages operator arrival and processing rates, scaling all operators in a single step by exploiting the streaming query topology and progressively computing the optimal per-operator parallelism. Dhalion [21] adopts a control-based approach using backpressure signals, such as load skew and slow instances, to detect resource contention and trigger scaling actions. DRS [23] applies queuing-theoretic models under steady-state assumptions to capture the impact of provisioned resources, providing a more structured framework for latency estimation than approaches based on backpressure or arrival rates. Finally, the Horizontal Pod Autoscaler (HPA) [1], the default Kubernetes autoscaling mechanism, scales deployments horizontally based on user-defined target metrics (e.g., average CPU or memory utilization). Styx currently does not support autoscaling policies; we leave this for future work.

## Future Work

**Stateful Serverless Architecture** State migration enables dynamic reconfiguration in Styx, enabling fully autonomous autoscaling. A key direction for future work is the design of an autoscaling mechanism that leverages runtime metrics to detect load imbalances and trigger resource rebalancing, allowing Styx to adapt transparently to workload changes in a serverless manner.

**Replication for High Availability** In the Styx architecture, replication is only applied in the snapshot store and the Input/Output queues to ensure fault tolerance. For high-availability, Styx could adopt replication mechanisms from deterministic databases. Specifically, the design of deterministic transaction protocols, such as Calvin [61], features state replicas that require no explicit synchronization. First, the sequencer replicas need to agree on the order of execution. After that, the deterministic sequencing algorithm guarantees that the resulting state will be the same across partition/worker replicas by all replicas executing state updates in the same order.

## Conclusion

This paper presented Styx, a distributed streaming dataflow system that supports multi-partition transactions with serializable isolation through a high-level Python programming model that abstracts away transaction failure handling. Styx integrates deterministic transaction execution with exactly-once dataflow processing, enabling end-to-end transactional guarantees across arbitrary function orchestrations. Experimental results show that Styx outperforms state-of-the-art systems by at least one order of magnitude in throughput. Moreover, Styx takes a step toward a serverless architecture by introducing a high-performance state migration mechanism that enables near-zero-downtime elasticity.

## References

[1] Kubernetes horizontal pod autoscaling. https://kubernetes.io/docs/tasks/run-application/horizontal-pod-autoscale/. Accessed: 2025-6-16

[2] Abadi, D.J., Faleiro, J.M.: An overview of deterministic database systems. Commun. ACM , (2018)

[3] Abebe, M., Glasbergen, B., Daudjee, K.: Morphosys: automatic physical design metamorphosis for distributed database systems. Proceedings of the VLDB Endowment **13**(13), 3573–3587 (2020)

[4] Armbrust, M., Das, T., Torres, J., Yavuz, B., Zhu, S., Xin, R., Ghodsi, A., Stoica, I., Zaharia, M.: Structured streaming: A declarative api for real-time applications in apache spark, p. SIGMOD. (2018)

[5] Beillahi, S.M., Bouajjani, A., Enea, C., Lahiri, S.: Automated synthesis of asynchronizations. In: International Static Analysis Symposium, pp. 135–159. Springer (2022)

[6] Burckhardt, S., Gillum, C., Justo, D., Kallas, K., McMahon, C., Meiklejohn, C.S.: Durable functions: semantics for stateful serverless. Proc. ACM Program. Lang. **5** (2021)

[7] Carbone, P., Ewen, S., Fóra, G., Haridi, S., Richter, S., Tzoumas, K.: State management in apache flink&reg;: Consistent stateful distributed stream processing. In: VLDB (2017)

[8] Carbone, P., Katsifodimos, A., Ewen, S., Markl, V., Haridi, S., Tzoumas, K.: Apache flink: Stream and batch processing in a single engine. Bulletin of the IEEE Computer Society Technical Committee on Data Engineering 36(4), (2015)

[9] Chandy, K.M., Lamport, L.: Distributed snapshots: determining global states of distributed systems, TOCS (1985)

[10] Chaoyi, C., Han, Mingzhe, H., Xu, N., Blanas, S., Bond, M.D., Wang, Y.: Developer’s responsibility or database’s responsibility? rethinking concurrency control in databases, CIDR (2023)

[11] Cheung, A., Crooks, N., Hellerstein, J.M., Milano, M.: New directions in cloud programming, CIDR (2021)

[12] Clark, C., Fraser, K., Hand, S., Hansen, J.G., Jul, E., Limpach, C., Pratt, I., Warfield, A.: Live migration of virtual machines. In: Proceedings of the 2nd conference on Symposium on Networked Systems Design & Implementation- **2**, 273–286 (2005)

[13] Collet, Y., Kucherawy, M.S.: Zstandard compression and the application/zstd” media type. RFC 8878, (2021)

[14] Cooper, B.F., Silberstein, A., Tam, E., Ramakrishnan, R., Sears, R.: Benchmarking cloud serving systems with ycsb. In: Proceedings of the 1st ACM symposium on Cloud computing, pp. 143–154. (2010)

[15] Das, S., Nishimura, S., Agrawal, D., Abbadi, A.E.: Albatross: Lightweight elasticity in shared storage databases for the cloud using live data migration. Proc. VLDB Endow. **4**(8), 494–505 (2011)

[16] Del Monte, B., Zeuch, S., Rabl, T., Markl, V.: Rhino: Efficient management of very large distributed state for stream processing engines. In: Proceedings of the 2020 ACM SIGMOD International Conference on Management of Data, (2020)

[17] Dey, A., Fekete, A., Nambiar, R., Röhm, U.: Ycsb+ t: Benchmarking web-scale transactional databases. In: 2014 IEEE 30th International Conference on Data Engineering Workshops, 223–230. IEEE (2014)

[18] Elmore, A.J., Arora, V., Taft, R., Pavlo, A., Agrawal, D., Abbadi, A.E.: Squall: Fine-grained live reconfiguration for partitioned main memory databases. In: Proceedings of the 2015 ACM SIGMOD International Conference on Management of Data, (2015)

[19] Elnozahy, E.N.M., Alvisi, L., Wang, Y.M., Johnson, D.B.: A survey of rollback-recovery protocols in message-passing systems. ACM Comput. Surv. 34(3), (2002)

[20] Fernandez, R.C., Migliavacca, M., Kalyvianaki, E., Pietzuch, P.: Making state explicit for imperative big data processing. In: USENIX annual technical conference (USENIX ATC 14), pp. 49–60. (2014)

[21] Floratou, A., Agrawal, A., Graham, B., Rao, S., Ramasamy, K.: Dhalion: self-regulating stream processing in heron. Proceedings of the VLDB Endowment **10**(12), 1825–1836 (2017)

[22] Fragkoulis, M., Carbone, P., Kalavri, V., Katsifodimos, A.: A survey on the evolution of stream processing systems. VLDB J. **33**(2), 507–541 (2024)

[23] Fu, T.Z.J., Ding, J., Ma, R.T.B., Winslett, M., Yang, Y., Zhang, Z.: DRS: auto-scaling for real-time stream analytics. IEEE/ACM Trans. Netw. **25**(6), 3338–3352 (2017)

[24] Gan, Y., Zhang, Y., Cheng, D., Shetty, A., Rathi, P., Katarki, N., Bruno, A., Hu, J., Ritchken, B., Jackson, B., et al.: An open-source benchmark suite for microservices and their hardware-software implications for cloud & edge systems. In: ASPLOS (2019)

[25] Gencer, C., Topolnik, M., Durina, V., Demirci, E., Kahveci, E.B., Lukás, A.G.O., Bartók, J., Gierlach, G., Hartman, F., Yilmaz, U., Dogan, M., Mandouh, M., Fragkoulis, M., Katsifodimos, A.: Hazelcast jet: Low-latency stream processing at the 99.99th percentile, VLDB (2021)

[26] Gjengset, J., Schwarzkopf, M., Behrens, J., Araújo, L.T., Ek, M., Kohler, E., Kaashoek, M.F., Morris, R.: Noria: dynamic, partially-stateful data-flow for high-performance web applications, p. OSDI. (2018)

[27] Gu, R., Yin, H., Zhong, W., Yuan, C., Huang, Y.: Meces: Latency-efficient rescaling via prioritized state migration for stateful distributed stream processing systems. In: 2022 USENIX Annual Technical Conference (USENIX ATC 22), pp. 539–556. (2022)

[28] Harenslak, B.P., de Ruiter, J.: Data Pipelines with Apache Airflow. Simon and Schuster (2021)

[29] de Heus, M., Psarakis, K., Fragkoulis, M., Katsifodimos, A.: Distributed transactions on serverless stateful functions. In: Proceedings of the 15th ACM International Conference on Distributed and Event-based Systems, 31–42 (2021)

[30] Hines, M.R., Deshpande, U., Gopalan, K.: Post-copy live migration of virtual machines. SIGOPS Oper. Syst. Rev. **43**(3), 14–26 (2009)

[31] Hoffmann, M., Lattuada, A., McSherry, F., Kalavri, V., Liagouris, J., Roscoe, T.: Megaphone: Latency-conscious state migration for distributed streaming dataflows. Proceedings of the VLDB Endowment **12**(9) (2019)

[32] Jacques-Silva, G., Zheng, F., Debrunner, D., Wu, K.L., Dogaru, V., Johnson, E., Spicer, M., Sariyüce, A.E.: Consistent regions: Guaranteed tuple processing in ibm streams, (2016)

[33] Jia, Z., Witchel, E.: Boki: Stateful serverless computing with shared logs. In: Proceedings of the ACM SIGOPS 28th Symposium on Operating Systems Principles, (2021)

[34] Jia, Z., Witchel, E.: Nightcore: efficient and scalable serverless computing for latency-sensitive, interactive microservices. In: Proceedings of the 26th ACM International Conference on Architectural Support for Programming Languages and Operating Systems, (2021) 10.1145/2451116.2451146 PMC3890416 24429939

[35] Kalavri, V., Liagouris, J., Hoffmann, M., Dimitrova, D., Forshaw, M., Roscoe, T.: Three steps is all you need: fast, accurate, automatic scaling decisions for distributed streaming dataflows. In: 13th USENIX Symposium on Operating Systems Design and Implementation, (2018)

[36] Killalea, T.: The hidden dividends of microservices. ACM Queue , (2016)

[37] Kreps, J., Narkhede, N., Rao, J.: Kafka: A distributed messaging system for log processing. In: Proceedings of the NetDB, vol. 11, pp. 1–7. (2011)

[38] Laigner, R., Zhou, Y., Salles, M.A.V., Liu, Y., Kalinowski, M.: Data management in microservices: State of the practice, challenges, and research directions. PVLDB 14(13), (2021)

[39] Lamport, L., Shostak, R., Pease, M.: The byzantine generals problem. ACM Trans. Program. Lang. Syst. **4**(3), 382–401 (1982)

[40] Lattuada, A., McSherry, F., Chothia, Z.: Faucet: a user-level, modular technique for flow control in dataflow engines. In: ACM SIGMOD BeyondMR Workshop. ACM (2016)

[41] Leutenegger, S.T., Dias, D.: A modeling study of the tpc-c benchmark. ACM SIGMOD Rec. **22**(2), 22–31 (1993)

[42] Li, T., Chandramouli, B., Burckhardt, S., Madden, S.: Darq matter binds everything: Performant and composable cloud programming via resilient steps. Proc. ACM Manag, Data (2023)

[43] Li, T., Chandramouli, B., Burckhardt, S., Madden, S.: Serverless state management systems, CIDR (2024)

[44] Lu, Y., Yu, X., Cao, L., Madden, S.: Aria: a fast and practical deterministic oltp database, VLDB (2020)

[45] Mao, Y., Junqueira, F.P., Marzullo, K.: Mencius: building efficient replicated state machines for wans. In: 8th USENIX Symposium on Operating Systems Design and Implementation (OSDI 08) (2008)

[46] Murray, D.G., McSherry, F., Isaacs, R., Isard, M., Barham, P., Abadi, M.: Naiad: a timely dataflow system. In: ACM SOSP (2013)

[47] Murray, D.G., Schwarzkopf, M., Smowton, C., Smith, S., Madhavapeddy, A., Hand, S.: CIEL: A universal execution engine for distributed Data-Flow computing. In: 8th USENIX Symposium on Networked Systems Design and Implementation (NSDI 11) (2011)

[48] Noghabi, S.A., Paramasivam, K., Pan, Y., Ramesh, N., Bringhurst, J., Gupta, I., Campbell, R.H.: Samza: Stateful scalable stream processing at linkedin, (2017)

[49] Psarakis, K., Christodoulou, G., Fragkoulis, M., Katsifodimos, A.: Transactional cloud applications go with the (data)flow. In: 15th Annual Conference on Innovative Data Systems Research (CIDR’25). January 19-22, 2025, Amsterdam, The Netherlands. (2025)

[50] Psarakis, K., Christodoulou, G., Siachamis, G., Fragkoulis, M., Katsifodimos, A.: Styx: Transactional stateful functions on streaming dataflows. Proc. ACM Manag, Data (2025)

[51] Psarakis, K., Zorgdrager, W., Fragkoulis, M., Salvaneschi, G., Katsifodimos, A.: Stateful entities: Object-oriented cloud applications as distributed dataflows. In: Proceedings 27th International Conference on Extending Database Technology, EDBT 2024 (2024)

[52] Qi, S., Liu, X., Jin, X.: Halfmoon: Log-optimal fault-tolerant stateful serverless computing. In: Proceedings of the 29th Symposium on Operating Systems Principles, pp. 314–330. (2023)

[53] Serafini, M., Taft, R., Elmore, A.J., Pavlo, A., Aboulnaga, A., Stonebraker, M.: Clay: fine-grained adaptive partitioning for general database schemas. Proceedings of the VLDB Endowment **10**(4), 445–456 (2016)

[54] Siachamis, G., Psarakis, K., Fragkoulis, M., van Deursen, A., Carbone, P., Katsifodimos, A.: Checkmate: Evaluating checkpointing protocols for streaming dataflows. In: 2024 IEEE 40th International Conference on Data Engineering (ICDE) (2024)

[55] Silvestre, P., Fragkoulis, M., Spinellis, D., Katsifodimos, A.: Clonos: Consistent causal recovery for highly-available streaming dataflows, p. SIGMOD. (2021)

[56] Skiadopoulos, A., Li, Q., Kraft, P., Kaffes, K., Hong, D., Mathew, S., Bestor, D., Cafarella, M., Gadepally, V., Graefe, G.: Dbos: a dbms-oriented operating system, (2021)

[57] Spenger, J., Carbone, P., Haller, P.: Portals: an extension of dataflow streaming for stateful serverless. In: Proceedings of the 2022 ACM SIGPLAN International Symposium on New Ideas, New Paradigms, and Reflections on Programming and Software, pp. 153–171. (2022)

[58] Sreekanti, V., Wu, C., Lin, X.C., Schleier-Smith, J., Gonzalez, J., Hellerstein, J.M., Tumanov, A.: Cloudburst: Stateful functions-as-a-service. Proc. VLDB Endow. **13**(11) (2020)

[59] Stonebraker, M., Çetintemel, U., Zdonik, S.: The 8 requirements of real-time stream processing. ACM SIGMOD Rec. **34**(4), 42–47 (2005)

[60] Thomson, A., Abadi, D.J.: The case for determinism in database systems, VLDB (2010)

[61] Thomson, A., Diamond, T., Weng, S.C., Ren, K., Shao, P., Abadi, D.J.: Calvin: fast distributed transactions for partitioned database systems. In: Proceedings of the 2012 ACM SIGMOD International Conference on Management of Data, pp. 1–12. (2012)

[62] Wang, S., Liang, E., Oakes, E., Hindman, B., Luan, F.S., Cheng, A., Stoica, I.: Ownership: A distributed futures system for Fine-Grained tasks. In: 18th USENIX Symposium on Networked Systems Design and Implementation, (2021)

[63] Wittig, A., Wittig, M.: Amazon Web Services in Action: An in-depth guide to AWS. Simon and Schuster (2023)

[64] Zhang, H., Cardoza, A., Chen, P.B., Angel, S., Liu, V.: Fault-tolerant and transactional stateful serverless workflows. In: 14th USENIX Symposium on Operating Systems Design and Implementation, (2020)

[65] Zhang, S., Soto, J., Markl, V.: A survey on transactional stream processing. VLDB J. 33(2), (2024)

